# Maternal exposure to buprenorphine, but not methadone, during pregnancy reduces social play behavior across two generations of offspring

**DOI:** 10.1007/s00213-024-06718-2

**Published:** 2024-12-05

**Authors:** Henriette Nyberg, Inger Lise Bogen, Egil Nygaard, Marijke Achterberg, Jannike Mørch Andersen

**Affiliations:** 1 Department of Forensic Sciences, Section of Forensic Research, Oslo University Hospital, PO Box 4950, Oslo, Norway; 2 Department of Pharmacy, Faculty of Mathematics and Natural Sciences, University of Oslo, Oslo, Norway; 3 Department of Psychology, PROMENTA, Faculty of Social Sciences, University of Oslo, Oslo, Norway; 4 Department of Population Health Sciences, Behavioral Neuroscience group, Faculty of Veterinary Medicine, University of Utrecht, Utrecht, The Netherlands

**Keywords:** Buprenorphine, Intergenerational, Medications for opioid use disorder, Methadone, Neurodevelopment, Prenatal exposure, Social behavior

## Abstract

**Rationale:**

The prevalence of newborns exposed to medications for opioid use disorder (MOUD), such as methadone or buprenorphine, during pregnancy is increasing. The opioid system plays a crucial role in regulating and shaping social behavior, and children prenatally exposed to opioids face an increased risk of developing behavioral problems. However, the impact of prenatal exposure to MOUD on offspring’s social behavior during adolescence and adulthood, as well as potential intergenerational effects, remains largely unexplored.

**Objectives:**

Our study employed a translationally relevant animal model to investigate how maternal (F0) exposure to MOUD during pregnancy affects social behavior in young and adult rats across the first (F1) and second (F2) generation of offspring.

**Methods:**

Female Sprague–Dawley rats were implanted with an osmotic minipump delivering methadone (10 mg/kg/day), buprenorphine (1 mg/kg/day), or sterile water, prior to mating with drug-naïve males. Adult F1 females were mated with treatment-matched F1 males to generate F2 offspring. We assessed social play behavior in juvenile offspring, and social interaction behavior in a three-chamber social interaction test in young adults of the F1 and F2 generations.

**Results:**

Maternal exposure to buprenorphine, but not methadone, during pregnancy reduced social play behavior in both F1 and F2 offspring, expressed by a reduced number of pounces and pins, which are the two most characteristic parameters of social play in rats. Adult social interactions were unaffected by prenatal MOUD exposure across both generations.

**Conclusions:**

Maternal exposure to buprenorphine during pregnancy may have adverse effects on social play behavior across two generations of offspring.

**Supplementary Information:**

The online version contains supplementary material available at 10.1007/s00213-024-06718-2.

## Introduction

The rising prevalence of legal and illicit opioid use has resulted in an increased number of children being exposed to these drugs during prenatal development (Bateman et al. 2014; Patrick et al. 2015). This is concerning, given that 85% of opioid-exposed children face various behavioral challenges as they age (Wilkinson et al. 2023), including emotional, cognitive and social deficits (Anand and Campbell-Yeo 2015; Ferguson et al. 2012; Harder and Murphy 2019; Hunt et al. 2008; Nygaard et al. 2015, 2016; Sandtorv et al. 2018; Spowart et al. 2023; Wilkinson et al. 2023; Yeoh et al. 2019), with indications of boys being more severely affected than girls (Nygaard et al. 2015; Skumlien et al. 2020). The recommended treatment of opioid dependence is medications for opioid use disorder (MOUD), such as methadone or buprenorphine, also during pregnancy (American Society of Addiction Medicine, 2020; American College of Obstetricians & Gynecologists, 2017). This treatment reduces maternal illicit drug use, improves prenatal care, and reduces maternal symptoms of craving and withdrawal (Minozzi et al. 2020). However, methadone and buprenorphine are exogenous opioids that cross the placenta (Kongstorp et al. 2019; Nanovskaya et al. 2008), and may interfere with fetal neurodevelopmental processes, resulting in adverse behavioral outcomes.

Children born to mothers receiving MOUD indeed exhibit significantly higher rates of attentional and emotional problems, as well as poorer language skills and lower cognitive test scores compared to unexposed children (Konijnenberg and Melinder 2015; Konijnenberg et al. 2016; Lee et al. 2019; Levine et al. 2021; Levine and Woodward 2018; Wouldes and Woodward 2020). Additionally, children exposed to MOUD have demonstrated visual-motor integration problems (Aslaksen et al. 2024) and lower scores on social maturity scales at 1.5 and 3 years of age (Hunt et al. 2008), as well as on social-emotional scales at 3 years (Salo et al. 2009). Children prenatally exposed to methadone also show increased rates of social withdrawal and higher scores on internalizing and externalizing behaviors in primary school age (6–13 years) (de Cubas and Field 1993). Despite these findings, clinical follow-up studies extending beyond early childhood are rare and encounter challenges due to various confounding factors that complicate the interpretation of the findings (Azuine et al. 2019; Handal et al. 2019).

Juvenile social play is a complex social behavior that is important for the development of social, cognitive and emotional skills by equipping the individual with a behavioral repertoire to face challenges in the environment (Pellis et al. 2010; Vanderschuren and Trezza 2014). Importantly, disrupted play experiences in young rats result in social and cognitive impairments, as well as long-lasting cellular and synaptic changes in prefrontal cortical neurons (Bijlsma et al. 2022; Baarendse et al. 2013). Pharmacological studies have demonstrated that the opioid system plays a major role in modulating social play behavior (Achterberg et al. 2019; Achterberg and Vanderschuren 2023; Manduca et al. 2014; Trezza and Vanderschuren 2008b). Specifically, acute systemic administration of µ-opioid receptor agonists prior to testing enhances social play in male and female juvenile rats (Manduca et al. 2014; Vanderschuren et al., 1995), an effect that is blocked by co-administration of µ-opioid receptor antagonists (Beatty and Costello, 1982; Trezza et al., 2011). Concerning exposure during development, a recent study has shown that morphine decreases play behavior in rats (Harder et al. 2023), whereas several older studies report increased play behavior following prenatal exposure to morphine (Buisman-Pijlman et al. 2009; Hol et al. 1996; Niesink et al. 1996, 1999). In terms of adult sociability, it has been demonstrated that rodents exposed to morphine during pregnancy exhibit impaired social interactions (Dunn et al. 2023; Smith et al. 2022, 2023). However, there is a notable knowledge gap regarding the impact of prenatal methadone or buprenorphine exposure on both juvenile and adult social behavior.

The identification of long-lasting effects in offspring prenatally exposed to opioids, raises the question of whether the impact of maternal (F0) opioid exposure during pregnancy may extend beyond the exposed fetus (F1), potentially influencing the health and behavior of subsequent generations (Yen and Davis 2022). The F2 generation could potentially be impacted by the opioid exposure either through alterations in the fetal germ cells of the F1 generation or by inheriting epigenetic changes (Gilardi et al. 2018). Preclinical studies have indeed demonstrated adverse effects in offspring of rats exposed to morphine during adolescence (Vassoler et al. 2016, 2017, 2019, 2022), but the intergenerational impact of opioid exposure during pregnancy on social behavior remains largely unexplored (Gilardi et al. 2018).

In the present study, we aimed to examine intergenerational effects of maternal methadone or buprenorphine exposure during gestation on juvenile play and adult social behavior in male and female offspring. We used a previously established MOUD exposure protocol in rats (Kongstorp et al. 2019, 2020) resulting in opioid blood concentrations in the dams similar to the concentrations reported in pregnant women receiving methadone or buprenorphine treatment (Concheiro et al. 2010; Welle-Strand et al. 2013). Our overall hypothesis was that maternal exposure to methadone or buprenorphine during pregnancy results in reduced social play behavior in juvenile offspring and impaired social interactions in adulthood across both the F1 and F2 generations.

## Materials and methods

### Animals and ethics statement

We obtained 59 male (12 weeks) and 59 female (16–18 weeks) Sprague–Dawley rats from Taconic Biosciences (Ejby, Denmark). Rats of the same sex were housed in groups of 3–4 per cage and allowed to acclimatize for 2 weeks. The rats had access to food and water ad libitum and were maintained on a 12 h light/dark cycle (lights on at 7 AM) in a controlled room with a temperature of 21 ± 2ºC and a humidity of 50 ± 10%. Experiments were conducted during the light phase of the day. All experimental procedures were conducted in compliance with the Norwegian Animal Welfare Act and the EU Directive 2010/63/EU, and were approved by the Norwegian Animal Research Authority (Norwegian Food Safety Authority, Oslo, Norway). The animals in the present study are part of a larger research project, i.e. all methods related to maternal exposure, mating and maternal separation are shared with other publications.

### Drugs

Methadone HCl (MW 345.91; Sigma, Oslo, Norway) and buprenorphine HCl (MW 504.11; Toronto Research Chemicals Inc, Toronto, Canada) were dissolved in sterile water (Fresenius Kabi, Hadeland, Norway). Sterile water was used as vehicle due to the low solubility of buprenorphine in saline.

### Maternal (F0) exposure and F1 breeding

**Fig. 1:**
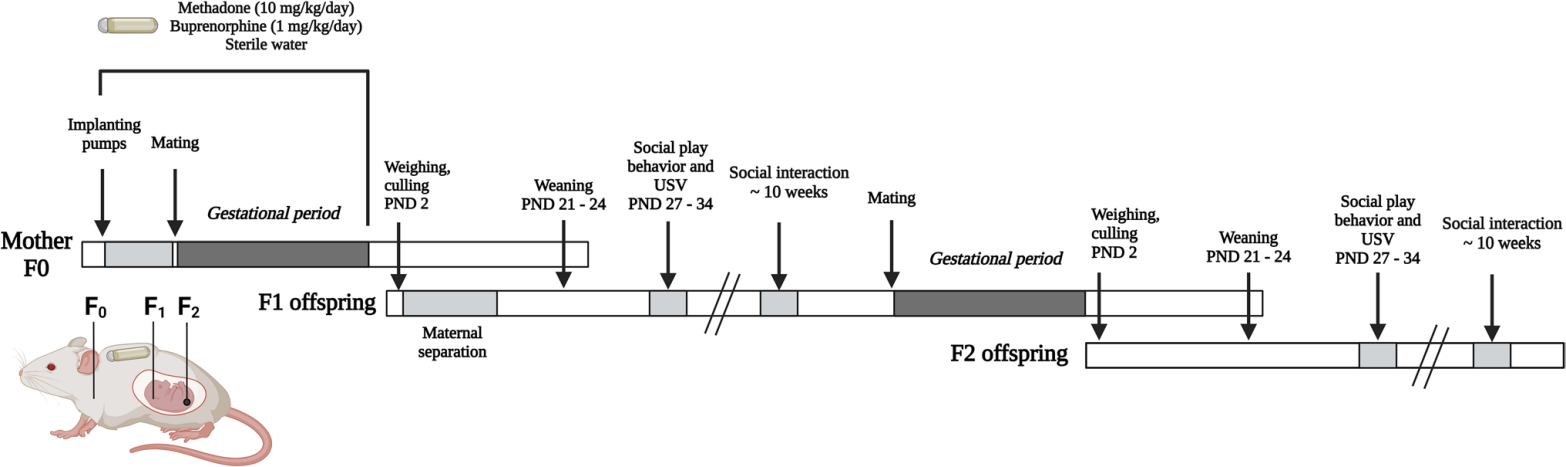
Experimental timeline. Female Sprague–Dawley rats (*n* = 16 – 22/treatment group) were implanted with a 28-day osmotic minipump filled with methadone (10 mg/kg/day), buprenorphine (1 mg/kg/day) or sterile water 4 days prior to mating. On postnatal day (PND) 2, the offspring were weighed and reduced to 8–10 pups in each litter, and on PND 21–24, the pups were weaned. The first 10 days after birth (PND 2–12), half of the F1 offspring in each litter underwent 3 h of daily maternal separation. On PND 27–34, social play behavior was examined and ultrasonic vocalization (USV) recorded. At approximately 10 weeks of age, the offspring’s social behavior was tested using a three-chamber social interaction test. After testing (15 weeks of age), F1 females were mated with treatment-matched F1 males to produce F2 offspring. The social behavior of the F2 offspring was investigated in the same manner as the F1 offspring. For more details, see the main text. Illustration created with BioRender.com

The 59 female rats (345 ± 3.6 g) were randomly assigned to one of three different treatment groups; methadone, buprenorphine or control. Compared to humans, methadone and buprenorphine have shorter half-lives in rats (Andersen et al. 2011; Ohtani et al. 1994). To mimic the human situation of stable blood concentrations throughout pregnancy, and to avoid the stress associated with repeated restraint and injections, the dams were implanted with a 28-day osmotic minipump (2ML4; Alzet, Cupertino, CA) filled with methadone (initial dose 10 mg/kg/day, n = 22), buprenorphine (initial dose 1 mg/kg/day, n = 21) or sterile water (n = 16) (Fig. 1). The doses were based on our previous work, demonstrating that 10 mg/kg/day methadone and 1 mg/kg/day buprenorphine result in opioid blood concentrations in the dams that are comparable to those observed in pregnant women receiving MOUD (Concheiro et al. 2010; Gordon et al. 2010; Kongstorp et al. 2019; Welle-Strand et al. 2013). The solutions were made on the day prior to implantation of the pumps. According to the manufacturer, all pumps are designed to administer substances for longer than the nominal duration of 28 days (Alzet). The pumps used in the present study had an administration duration of 31.4 days, calculated based on the fill volume and lot-specific flow rate. The average duration from pump implantation to birth was 27.3 ± 0.27 days (mean ± SEM) for control dams, 27.4 ± 0.30 days for methadone-exposed dams and 27.0 ± 0.27 days for buprenorphine-exposed dams, with a maximum of 29 days across all treatment groups. This demonstrates that the pups were continuously exposed to their respective substance throughout gestation. The pumps were implanted under isoflurane anesthesia (Baxter International, Deerfield, IL). A small lateral incision was made in the dorsal area behind the neck and the pump was inserted subcutaneously (s.c.) with the opening facing the anterior direction. The incision site was closed with nonabsorbable stitches. The animals were allowed to recover on a heating pad under observation before they were placed in individual cages. Marcaine (1 mg/kg, s.c., Aspen Pharma Trading, Dublin, Ireland) and Metacam (0.3 mg/kg, s.c., Boehringer Ingelheim Vetmedica GmbH, Copenhagen, Denmark) were given as analgesics during the surgery. Metacam was also given 4–5 h after the surgery. After implantation, the females were allowed to recover for 3–4 days before they were co-housed with a drug-naïve male for 1 week to facilitate mating. After one week, the females (F0) were single housed for the rest of the gestational period, and together with the offspring (F1) until weaning.

As the pump was estimated to be empty a few days after birth, and a newborn rat is considered to be comparable to a human fetus at late second to early third trimester (Semple et al. 2013), the pups in the current study were exposed to opioids in the period corresponding to the first and second trimester of a human pregnancy. Discontinuation of exposure could potentially result in withdrawal symptoms in both the dam and the offspring. However, we have previously reported that maternal care appears to be unaffected by this treatment protocol, and that no apparent signs of withdrawal are present in the pups (Kongstorp et al. 2020).

Two vehicle-exposed and one methadone-exposed animal died during labor, while three methadone-exposed and one buprenorphine-exposed were euthanized during pregnancy due to health problems or complications with the pump. In addition, three methadone- and three buprenorphine-exposed females did not mate successfully. One buprenorphine-exposed dam consumed all her pups on postnatal day (PND) 0–1. Therefore, a total of 45 females (14 control, 15 methadone, and 16 buprenorphine) were included as mothers to the F1 offspring. These mothers and offspring were also included in another publication studying anxiety-like behavior following prenatal exposure to MOUD (Nyberg et al. 2024).

### F2 breeding

After the F1 offspring had undergone the behavioral testing and reached 15 weeks of age, they were used to breed the F2 generation. A total of 36 female and 36 male F1 rats, divided into 12 control pairs, 11 methadone pairs, and 13 buprenorphine pairs, were used for breeding. Each female was mated with a treatment-matched male from a different litter for one week. After mating, the females (F1) were single housed for the rest of the gestational period, and together with the offspring (F2) until weaning. Due to health problems after birth, one methadone-exposed and one vehicle-exposed F1 female were euthanized along with their pups. One methadone and two buprenorphine-exposed F1 females did not mate successfully, and one buprenorphine-exposed F1 dam consumed all her pups on PND 0–1. Thus, a total number of 11 control pairs, 9 methadone pairs and 10 buprenorphine pairs served as parents to the F2 offspring.

### Offspring (F1 and F2)

The day of birth was designated as PND 0. To ensure that each dam raised approximately the same number of pups, all litters were culled to 8–10 pups on PND 2 with as equal numbers of males and females as possible. The offspring were earmarked and weaned on PND 21–24. After weaning, the rats were housed in groups of 2–4 of the same sex in each cage, with the same housing conditions as described in section “Animals and ethics statement”. A total of 518 F1 offspring and 330 F2 offspring were included in the present study.

From PND 2 to PND 12, half of the pups in each litter of the F1 generation were separated from the dam for 3 h per day (9 – 12 AM). The separated pups were placed together in groups of 2–5 littermates in a Plexiglas box (20 × 20 cm) with wood shavings. The box was placed on a heating pad in a quiet room with dimmed light conditions. The dam and the remaining littermates were placed together in the holding room. After 3 h of separation, the pups were returned to their home cage with their dam and non-separated littermates. To ensure consistent treatment each day, all pups were marked daily with a non-toxic skin marker. In the present study, statistical analyses revealed that maternal separation did not affect any of the outcomes examined, and the data were therefore combined across maternal separation status.

### Behavioral testing of F1 and F2 offspring

#### Social play behavior and ultrasonic vocalization

On PND 27 – 34, social play behavior was investigated as previously described (Achterberg et al. 2014). The test arena was a Plexiglas cage (40 × 40 × 50 cm) with approximately 3 cm of wood shavings covering the floor. The testing was performed in a quiet room under dimmed light conditions (< 5 lx).

In the two days prior to testing, the rats were individually habituated to the test cage for 10 min each day to minimize the influence of the novel test environment on the expression of social play behavior. On the test day, the animals were socially isolated in separate cages for 3 h prior to testing to enhance their social motivation and thus facilitate the expression of social play (Panksepp and Beatty 1980). After isolation, each rat was paired with an unfamiliar sex- and age-matched partner from another home cage and placed together in the test cage for 15 min. Since social play behavior in rats strongly depends on the playfulness of its partner (Pellis et al. 2022; Trezza and Vanderschuren 2008a), both rats in a pair were from the same treatment group and did not differ more than 10 g in body weight.

The play behavior was recorded using a camera (Basler acA1300 – 60gmNIR) connected to the Ethovision XT 13/16 software (Noldus Information Technology, Wageningen, The Netherlands), and then manually assessed using the Observer XT 15/16 software (Noldus Information Technology, Wageningen, The Netherlands) by an experienced observer blinded to the treatment groups. Play behavior was assessed per pair of animals. The number of the play-related behaviors pounces, pins and boxes were quantified, as well as the time spent on social and non-social exploration (Table 1).

**Table 1:** Description of recorded behaviors during social play testing. Adapted from Trezza et al. (2010)

Behaviors related to social play	
Pouncing	One rat approaches the conspecific and attempts to nose or rub the nape of its neck. This is considered an invitation to play
Pinning	The rat that is pounced upon fully rotates to its dorsal surface with the other rat standing over it. This is considered to continue the play interaction
Boxing	The rat that is pounced upon rears towards the soliciting rat and the two rats rapidly push, paw, and grab each other
Behaviors unrelated to social play	
Social exploration	The total time spent in non-playful forms of social interaction (i.e. sniffing or grooming any part of the partner’s body)
Non-social exploration	The total time spent exploring the cage or walking around

During the play session, ultrasonic vocalization (USV) was recorded using the UltraVox 3.0 Ultrasound Microphone connected to the UltraVox 3.0 software (Noldus Information Technology, Wageningen, The Netherlands), with a sampling rate of 250.000 Hz (recording range: 0–125 kHz; 16 Bit). The microphone was placed 20 cm above the floor of the behavior chamber. The recordings were transferred to Raven Pro 1.6 software (Bioacoustics Research Program, Cornell Lab of Ornithology, Ithaca, NY) for acoustical analysis by a researcher blinded to the treatment groups. As the amount of play is most prominent in the beginning of a play session, all calls with a frequency between 20 and 80 kHz during the first 5 min were manually counted and classified (Table 2), according to the modified protocol by Wright et al. (2010). See Fig. S1 for examples of different types of calls. Calls were assessed per pair of animals. Of note, 22-kHz calls, indicating negative affect, were only observed in 2 out of 80 pairs of animals and these vocalizations were not included in the analyses.

**Table 2:** Classification of 50-kHz ultrasonic vocalizations (USVs) in the present study and in the study by Wright et al. (2010)

Present study	Wright et al. (2010)
Trill	Trills, trills with jumps, flat-trills
Step	Step-up, step-down, multi-step, split
Short	Short
Flat	Flat
Other	Complex, upward ramp, downward ramp, inverted u, composite

#### Three-chamber social interaction test

At 10 weeks of age, the offspring were tested in the three-chamber social interaction test, also known as Crawley’s sociability test (Servadio et al. 2016). The apparatus was a Plexiglas box (117 × 77 × 40 cm; Noldus Information Technology) divided into three equally sized compartments (left, center, right; each 38 × 77 cm) by Plexiglas walls with openings (10 × 11 cm). The left and right chambers contained wire cages (d = 22 cm) in the center of the compartment that were used to hold novel rats. The novel rats were unfamiliar to the rat being tested, but similar in age, sex and weight.

The test consisted of three phases; habituation (5 min), test of sociability (10 min), and test of social novelty (10 min). In the habituation phase, the rat was placed in the center chamber with the opening to the left and right chambers closed. In the sociability phase, a novel same-sex conspecific was placed in one of the wire cages in the side chamber, serving as a 'social stimulus', while the empty wire cage located in the other side chamber served as a 'non-social stimulus'. The dividers were raised, allowing the rat to move freely throughout all three chambers of the apparatus. In the social novelty phase, a second novel same-sex conspecific was placed in the empty wire cage. The conspecific introduced in the sociability phase now served as the 'familiar stimulus', while the novel conspecific served as the 'novel stimulus'. The sides for the familiar versus the novel rat were counterbalanced. The trials were video recorded with a camera (Basler acA1300 – 60gmNIR) and tracked with Ethovision version 13/16 (Noldus Information Technology, Wageningen, The Netherlands). The time spent in the different chambers during the sociability phase and the social novelty phase was determined, and used to calculate the sociability index and social novelty index:

$$Sociability\;Index=\frac{Time\;social\;stimulus}{Time\;social\;stimulus+Time\;non\text{-}social\;stimulus}$$

$$Social\;Novelty\;Index=\frac{Time\;novel\;stimulus}{Time\;novel\;stimulus+Time\;familiar\;stimulus}$$

##### Statistical analyses

The statistical analyses were performed in R (version 4.3.0). One-way ANOVA followed by Dunnett’s post hoc test was used to analyze litter size and sex ratio per litter. Chi-squared tests were used to analyze sex ratio. Mixed effects regressions, estimated using restricted maximum likelihood (REML) and implemented in the ‘lme4’ package in R, were used to analyze all outcome variables where each pup served as the statistical unit, which included all behavioral parameters as well as weight data. The reasoning for using mixed effects models is their ability to easily include covariates at both within- and between subject levels, as well as different random effect variables. Additionally, mixed models are flexible with regard to unequal group sizes and missing data.

All models included the fixed effects treatment group (factor with three levels), offspring sex (dichotomous factor), maternal separation status (dichotomous factor, only included for the F1 generation), and treatment-by-sex and treatment-by-maternal separation (only included for the F1 generation) interactions. A random intercept for batch was included in all models to account for the non-independence of data if the intraclass correlation coefficient (ICC) exceeded 0.1 in the null model with no fixed effects (outcome variable ~ 1 + (1|Batch)). A random intercept for litter was included in the models used to analyze weight data and social interaction data, but not in the models analyzing social play data and USV, as the unit consisted of two animals from different litters. ICCs for null models and full models are reported in Table S1 and S2. As maternal separation did not have a significant main effect, nor were involved in any interactions, results were combined across maternal separation status.

The distribution of the residuals was inspected by use of Q-Q plots and histograms, and found satisfactory. Contrast comparisons of *a priori* specified groups were performed using the package ‘emmeans’ with the Kenward-Roger degrees-of-freedom method and the Dunnett’s correction for multiple comparisons. A *p* ≤ 0.05 was considered statistically significant. Data are presented as the mean ± SEM with data points depicted in the bar charts. Each data point represents a pair of rats for social play behavior and USV, and one individual rat for social interaction data.

## Results

### Litter characteristics and neonatal outcomes

The litter characteristics and neonatal outcomes for the F1 generation have previously been reported in Nyberg et al. (2024), but are included here for comparison with the F2 generation. In short, prenatal exposure to methadone or buprenorphine reduced the number of F1 offspring per litter (Table 3). The sex ratio per litter was significantly skewed towards more males in the methadone-exposed group, while no such skewness was observed for total sex ratio (Table 3). In addition, exposure to methadone reduced body weight on PND 2 in both male and female pups, compared to controls (Table 4). In the F2 generation, there was no significant effect of parental methadone or buprenorphine exposure on the number of offspring per litter, sex ratio (Table 3), or the body weight on PND 2 or PND 21 (Table 4). At 10 weeks of age, male pups born by parents exposed to buprenorphine *in utero* had a lower body weight compared to the controls (Table 4).

**Table 3:** Effects of maternal exposure (F0) to methadone (10 mg/kg/day) or buprenorphine (1 mg/kg/day) on litter characteristics in the F1 and F2 generations

	Control	Methadone	Buprenorphine
F1 generation #			
Number of dams	14	15	16
Number of offspring per litter	14.57 ± 0.48	9.73 ± 0.67***	10.50 ± 0.80***
Total sex ratio (males/females)	89/114	76/70	82/87
Sex ratio per litter (males/females)	0.81 ± 0.09	1.27 ± 0.16*	1.03 ± 0.12
F2 generation †			
Number of dams	12	10	10
Number of offspring per litter	10.42 ± 0.86	10.50 ± 1.10	10.00 ± 0.52
Total sex ratio (males/females)	63/61	44/60	50/50
Sex ratio per litter (males/females)	1.17 ± 0.22	0.77 ± 0.11	1.17 ± 0.25

Statistical analyses of the number of offspring and sex ratio per litter were performed with a one-way ANOVA, followed by Dunnett’s post hoc test. Statistical analysis of total sex ratio was performed with a chi-squared test. †During breeding of the F2 generation, one control dam and one methadone-exposed dam, along with their offspring, were euthanized shortly after birth due to health complications. *p < 0.05, ***p < 0.001, compared to control. The number of offspring and the sex ratio per litter are presented as mean ± SEM. #Reported in Nyberg et al. (2024)

**Table 4:** Effects of maternal exposure (F0) to methadone (10 mg/kg/day) or buprenorphine (1 mg/kg/day) on body weight in the F1 and F2 generations

	Control	Methadone	Buprenorphine
**F1 generation #**			
Body weight PND 2 (g)			
Males + females	8.43 ± 0.07	7.44 ± 0.08**	8.64 ± 0.09
Males	8.65 ± 0.11	7.70 ± 0.12**	8.92 ± 0.12
Females	8.26 ± 0.08	7.16 ± 0.11*	8.38 ± 0.12
Body weight PND 21 (g)			
Males + females	59.49 ± 1.25	64.53 ± 1.47	62.48 ± 1.23
Males	60.37 ± 1.88	65.75 ± 1.88	63.45 ± 1.80
Females	58.78 ± 1.69	63.08 ± 2.33	61.57 ± 1.70
Body weight 10 weeks (g)			
Males + females	289.84 ± 7.75	297.89 ± 9.08	277.03 ± 8.01
Males	346.47 ± 6.26	358.14 ± 6.81	331.72 ± 8.08
Females	233.21 ± 3.25	235.50 ± 3.85	225.87 ± 2.53
**F2 generation**			
Body weight PND 2 (g)			
Males + females	8.57 ± 0.11	8.16 ± 0.08	8.80 ± 0.10
Males	8.84 ± 0.17	8.46 ± 0.11	9.00 ± 0.15
Females	8.30 ± 0.11	7.94 ± 0.11	8.59 ± 0.14
Body weight PND 21 (g)			
Males + females	62.18 ± 1.16	60.37 ± 1.21	62.27 ± 1.23
Males	63.67 ± 1.79	61.75 ± 1.91	64.29 ± 1.89
Females	60.76 ± 1.49	59.31 ± 1.55	60.34 ± 1.56
Body weight 10 weeks (g)			
Males + females	315.78 ± 10.99	304.28 ± 11.22	295.28 ± 10.62
Males	383.09 ± 6.93	371.40 ± 6.79	354.67 ± 7.61*
Females	242.62 ± 3.77	237.17 ± 4.41	235.88 ± 3.70

Statistical analyses of body weight were performed with a LMM with treatment, sex and treatment*sex as fixed factors. Litter and batch were included as random factors when the ICC > 0.1. Body weight PND 2, *n* = 70 –115 (F1) and *n* = 44 – 60 (F2) per treatment group/sex. Body weight PND 21, *n* = 30 – 47 (F1) and *n* = 30 – 44 (F2) per treatment group/sex. Body weight 10 weeks, *n* = 28 – 34 (F1) and *n* = 19 – 25 (F2) per treatment group/sex. *p < 0.05 **p < 0.01, compared to control. Data are shown as mean ± SEM. #Reported in Nyberg et al. (2024). Abbreviations. *ICC* intraclass correlation coefficient; *LMM* linear mixed model; *PND* postnatal day

### Effects of maternal methadone or buprenorphine exposure on social play behavior and ultrasonic vocalization

To determine the generational impact of maternal exposure to methadone or buprenorphine on juvenile social play behavior, we investigated different metrics of play in both F1 and F2 offspring. Maternal separation of the F1 offspring did not affect any of the play parameters tested, hence, the data were collapsed on maternal separation status. Statistical results of the overall effects on social play behavior in the F1 and F2 generations are shown in Table S3 and S4, with pairwise comparisons (treatment vs. control within each sex) available in Table S5 and S6. Statistical results of main effects and pairwise comparisons for ultrasonic vocalization are found in Tables S7 – S10.

#### Social play behavior in the F1 generation

**Fig. 2:**
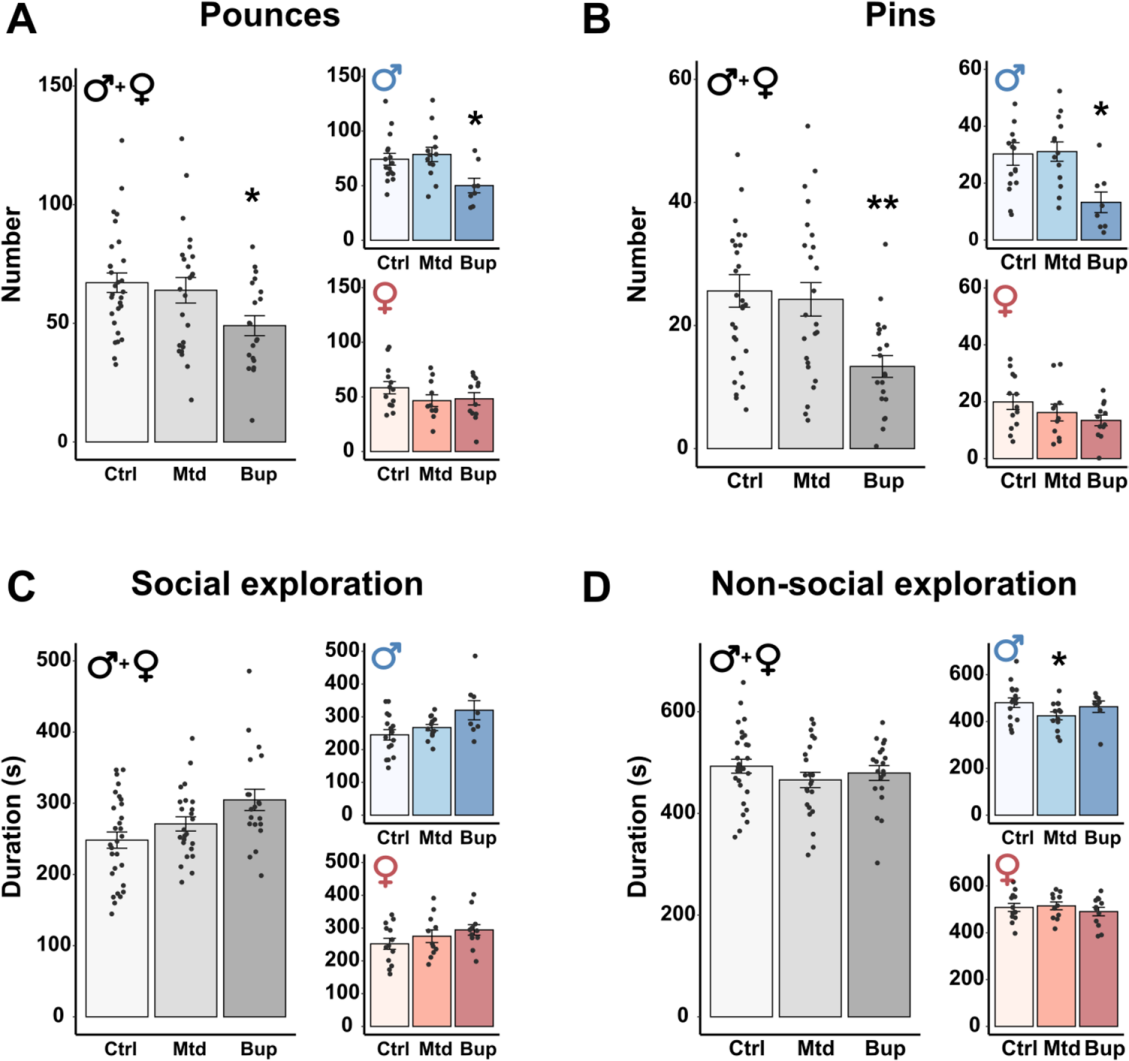
Social play behavior in the F1 generation. Number of pounces (**A**) and pins (**B**), and duration (s) of social exploration (**C**) and non-social exploration (**D**) in male and female offspring exposed to methadone (10 mg/kg/day) or buprenorphine (1 mg/kg/day) *in utero*. Males and females combined are shown in grey-scale, while separate results for the sexes are shown in blue (males) and red (females). All data are collapsed across maternal separation status. Values are shown as mean ± SEM, along with data points representing pairs of rats, *n* = 8–15 pairs/treatment group/sex. *p < 0.05 **p < 0.01, compared to control. Abbreviations. Bup, buprenorphine; Ctrl, control; Mtd, methadone

There was an overall effect of treatment on the number of pounces (F2,64 = 4.417; *p* = 0.016) and pins (F2,64 = 6.860; *p* = 0.002). Pairwise comparisons revealed that the F1 offspring prenatally exposed to buprenorphine pounced and pinned significantly less compared to the animals in the control group (Fig. 2A-B, Males + Females). There were no overall effects of treatment on social or non-social exploration (Table S3). There was an overall effect of sex for pouncing (F1,64 = 11.532; *p* = 0.001) and pinning (F1,64 = 9.172; *p* = 0.004), as well as the duration of non-social exploration (F1,64 = 9.269; *p* = 0.003), where females pounced and pinned less and spent more time on non-social exploration compared to males.

There was an interaction effect between treatment and sex for the number of pounces (F2,64 = 3.084; *p* = 0.053). Pairwise comparisons showed that F1 males prenatally exposed to buprenorphine pounced and pinned less than animals in the control group (Fig. 2A-B, Males). The same direction of effect was observed for buprenorphine-exposed females when compared to controls, although not significant. Furthermore, the pairwise comparisons also revealed that methadone-exposed males, but not females, spent significantly less time on non-social exploration compared to controls (Fig. 2D, Males). There was an overall effect of treatment on the number of boxes, with buprenorphine-exposed offspring boxing less than controls (Fig. S2A), but no effect of treatment on the latency to first pounce or first pin (Fig. S2B-C).

#### Ultrasonic vocalization in the F1 generation

**Fig. 3:**
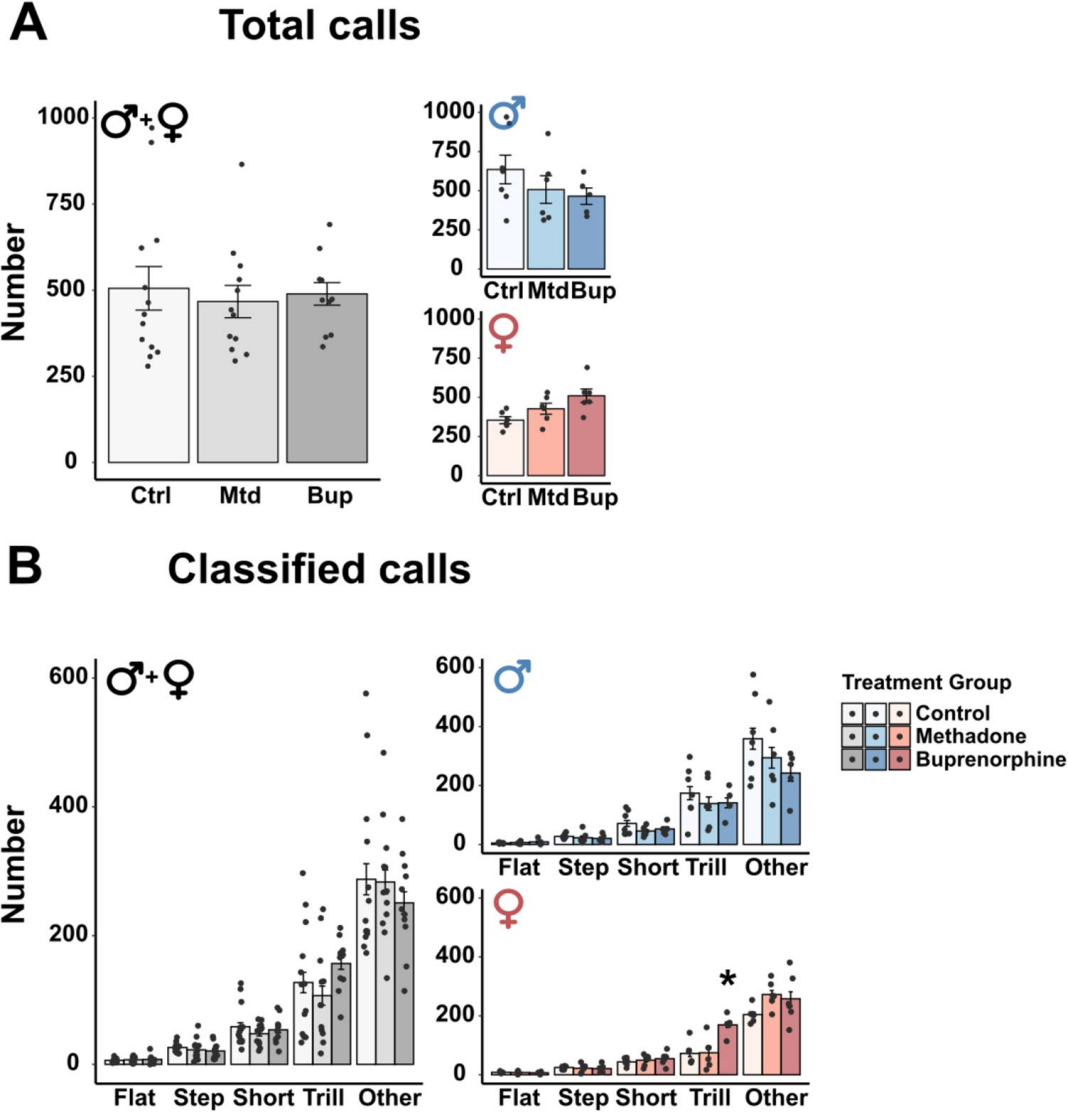
Ultrasonic vocalizations during social play behavior in the F1 generation. Number of total calls (**A**) and classified calls (**B**) in male and female offspring exposed to methadone (10 mg/kg/day) or buprenorphine (1 mg/kg/day) *in utero*. Males and females combined are shown in grey-scale, while separate results for the sexes are shown in blue (males) and red (females). All data are collapsed across maternal separation status. Values are shown as mean ± SEM, along with data points representing pairs of rats, *n* = 5–7 pairs/treatment group/sex. *p < 0.05, compared to control. Abbreviations. Bup, buprenorphine; Ctrl, control; Mtd, methadone

The data on USV during social play revealed no overall effect of treatment on the total number of calls or on any of the call types (Table S7). An interaction effect between treatment and sex were found for the total number of calls (Table S7), but the pairwise comparisons revealed no significant effect of treatment within each sex (Fig. 3A). There was also a significant interaction between treatment and sex for the number of emitted trill calls (Table S7) and pairwise comparisons revealed that F1 females prenatally exposed to buprenorphine emitted more trill calls compared to controls (Fig. 3B, Females). There was a main effect of sex for the total number of calls and for the number of emitted trills (Table S7), where males vocalized more than females.

#### Social play behavior in the F2 generation

**Fig. 4:**
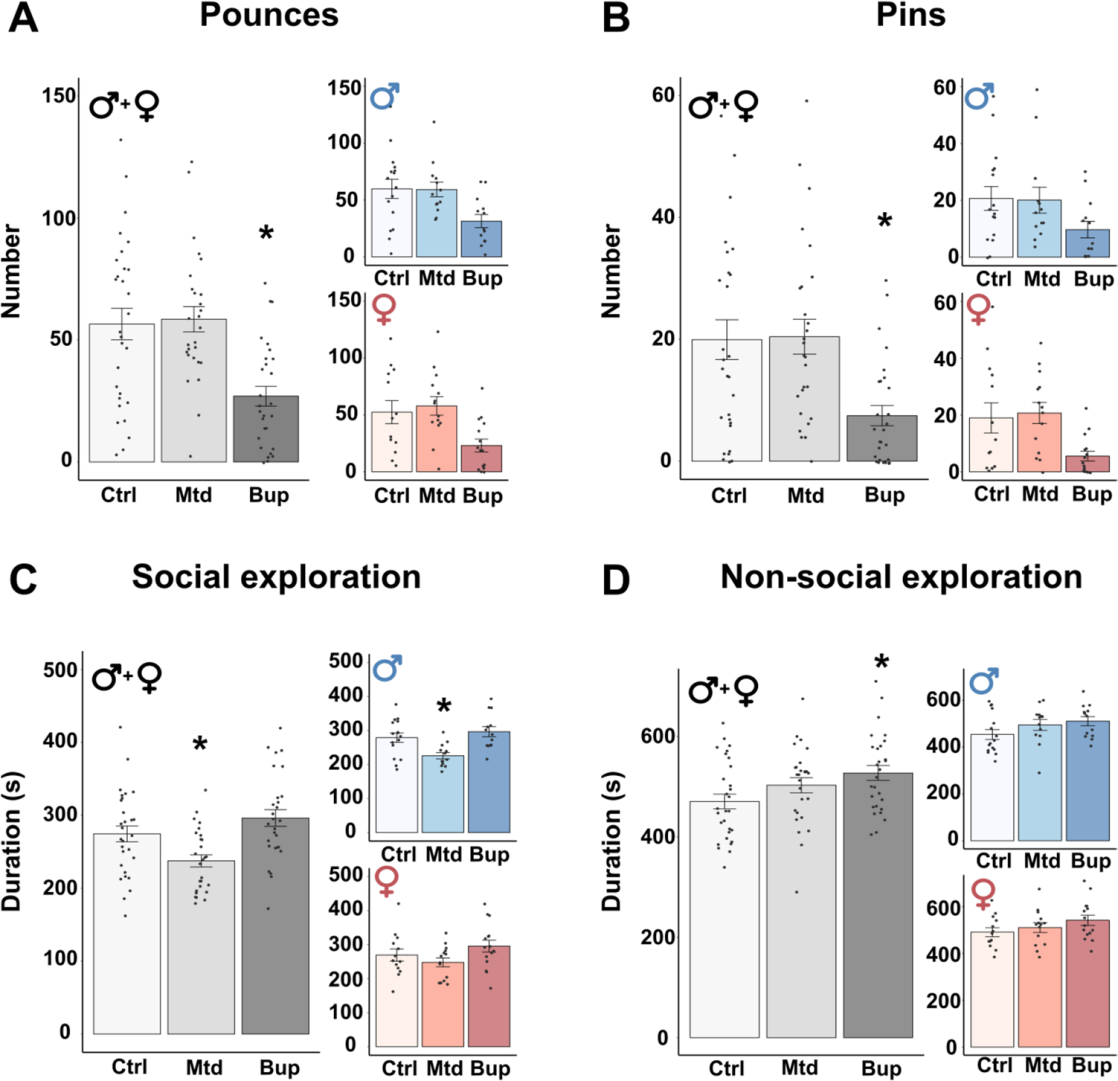
Social play behavior in the F2 generation. Number of pounces (**A**) and pins (**B**), and duration (s) of social exploration (**C**) and non-social exploration (**D**) in male and female offspring born to parents exposed to methadone (10 mg/kg/day) or buprenorphine (1 mg/kg/day) *in utero*. Males and females combined are shown in grey-scale, while separate results for the sexes are shown in blue (males) and red (females). Values are shown as mean ± SEM, along with data points representing pairs of rats, *n* = 13 – 16 pairs/treatment group/sex. *p < 0.05, compared to control. Abbreviations. Bup, buprenorphine; Ctrl, control; Mtd, methadone

Similar to the F1 generation, the F2 generation had an overall effect of treatment on the number of pounces (F2,76.02 = 5.875; *p* = 0.004) and pins (F2,76.03 = 4.480; *p* = 0.015), and also on the duration spent on social (F2,75.09 = 4.596; *p* = 0.013) and non-social exploration (F2,76.01 = 4.155; *p* = 0.019). Pairwise comparisons revealed that F2 animals in the buprenorphine group pounced and pinned less (Fig. 4A-B, Males + Females), and spent more time on non-social exploration (Fig. 4D, Males + Females) compared to the control group. Animals born to parents prenatally exposed to methadone spent less time on social exploration compared to animals in the control group (Fig. 4C, Males + Females), with a significant reduction for males (Fig. 4C, Males). Sex had an overall effect on the duration spent on non-social exploration (F1,76.10 = 5.199; *p* = 0.025), with females spending more time on non-social exploration compared to males. There were no interaction effects between treatment and sex (Table S4). There was an effect of treatment on the number of boxes and the latency to first pin, where animals in the buprenorphine group boxed less (Fig. S3A) and had a longer duration until the first pin (Fig. S3C) compared to animals in the control group. There was no effect of treatment on the latency to first pounce (Fig. S3B).

#### Ultrasonic vocalization in the F2 generation

**Fig. 5:**
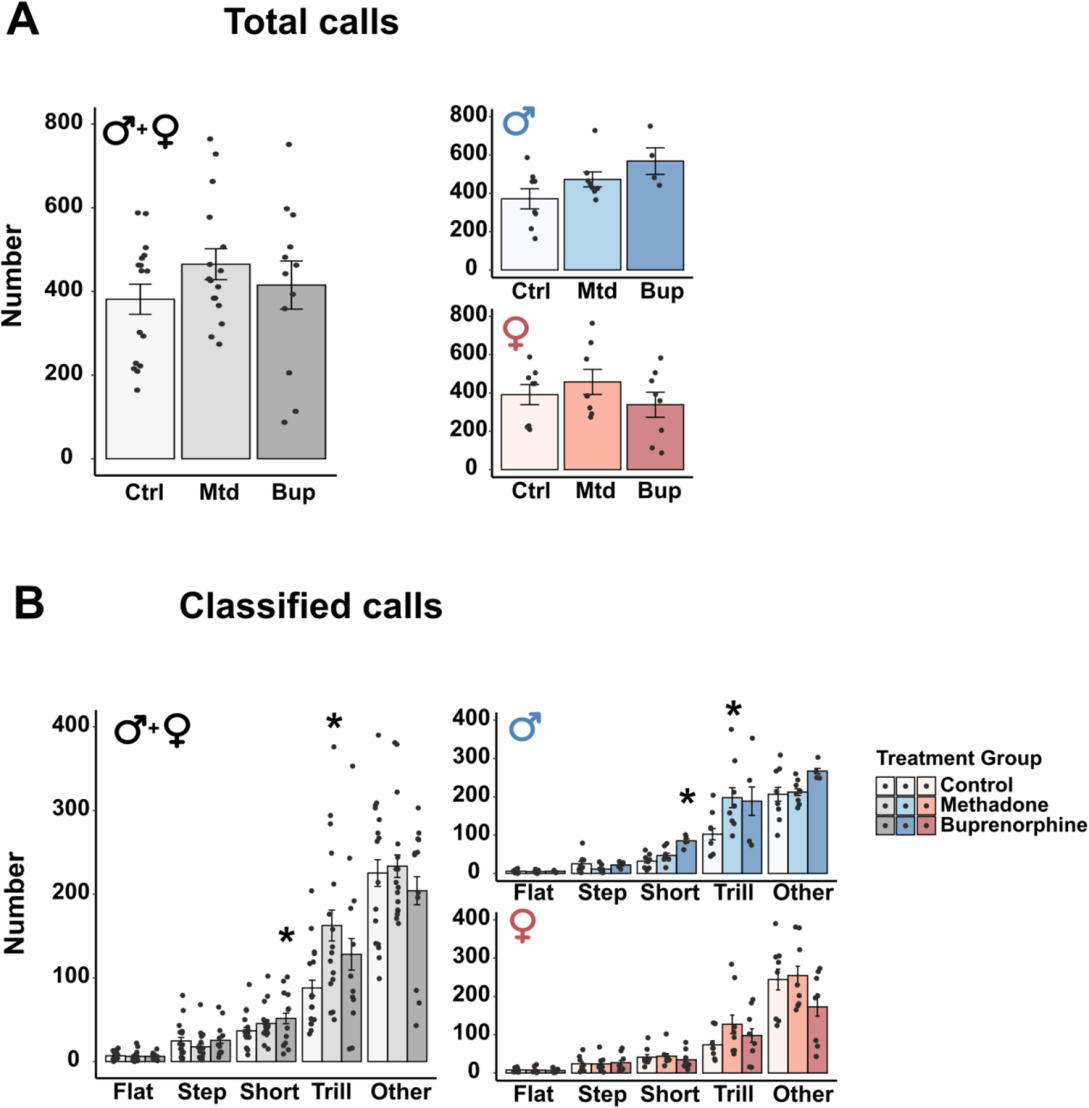
Ultrasonic vocalizations during social play behavior in the F2 generation. Number of total calls (**A**) and classified calls (**B**) in male and female offspring born to parents exposed to methadone (10 mg/kg/day) or buprenorphine (1 mg/kg/day) *in utero*. Males and females combined are shown in grey-scale, while separate results for the sexes are shown in blue (males) and red (females). Values are shown as mean ± SEM, along with data points representing pairs of rats, *n* = 4 – 8 pairs/treatment group/sex. *p < 0.05, compared to control. Abbreviations. Bup, buprenorphine; Ctrl, control; Mtd, methadone

Regarding USV during social play, there was no overall effects on the total number of calls (Table S9). When analyzing the different call types separately, there was an overall effect of treatment on the number of emitted trill calls and short calls (Table S9). Pairwise comparisons revealed that F2 animals in the methadone group emitted more trill calls, whereas animals in the buprenorphine group emitted more short calls compared to control animals (Fig. 5B, Males + Females). There was an interaction effect between treatment and sex for calls classified as other and short (Table S9). Pairwise comparisons revealed no significant differences for calls classified as other, but males in the buprenorphine group emitted more short calls compared to controls (Fig. 5B, Males). Moreover, males in the methadone group emitted more trill calls compared to controls (Fig. 5B, Males). There was a main effect of sex for the number of emitted trill calls and short calls (Table S9), where males vocalized more than females.

### Effects of maternal methadone or buprenorphine exposure on sociability and social novelty preference

We investigated social behavior in 10-week-old rats in both the F1 and F2 generations, using a three-chamber social interaction test. Maternal separation of the F1 offspring did not affect any of the parameters tested, hence, the data were collapsed on maternal separation status.

**Fig. 6:**
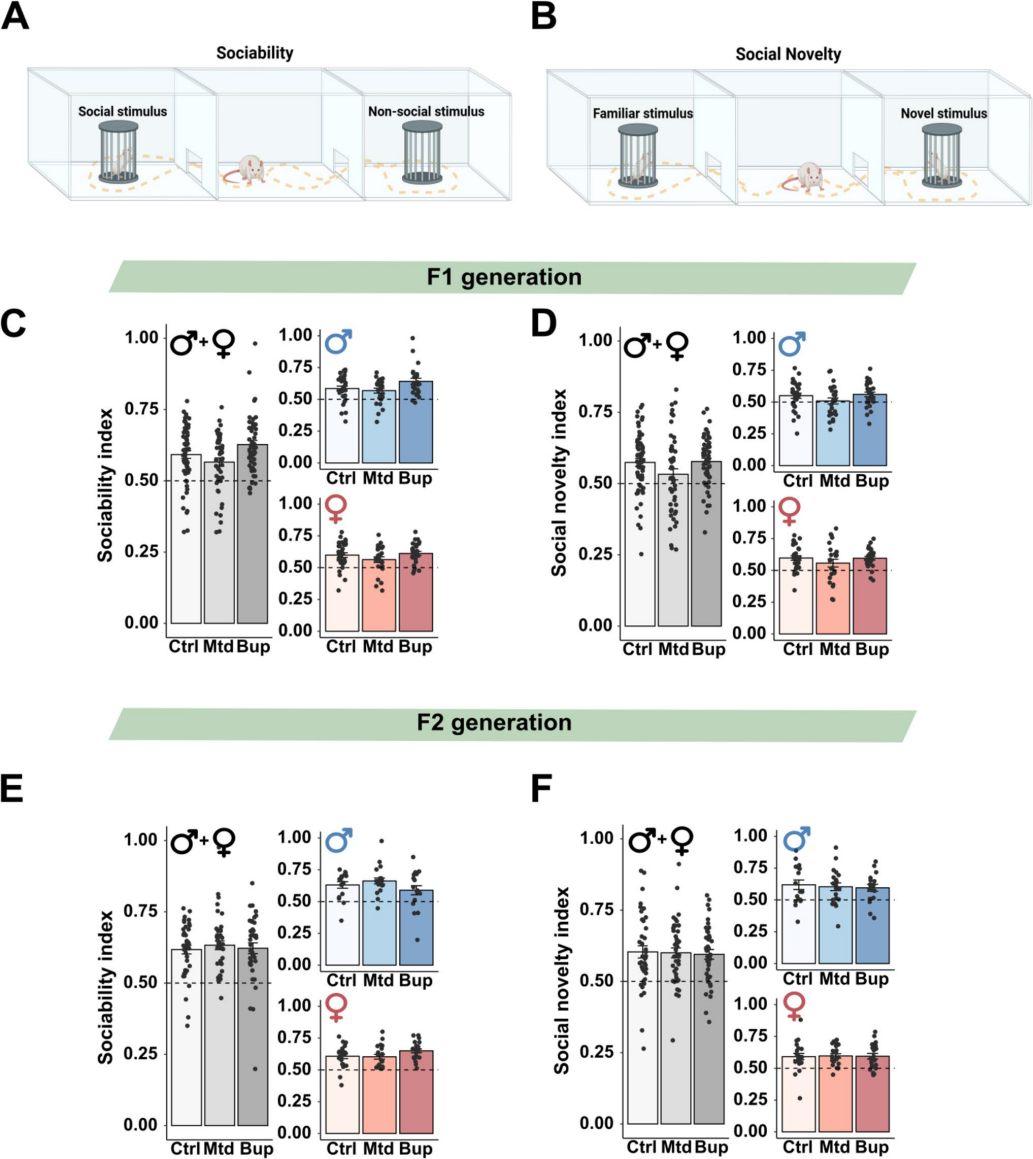
Three-chamber social interaction test in the F1 and F2 generations. Illustration of the sociability phase (**A**) and the social novelty phase (**B**). The sociability index and social novelty index for male and female offspring in the F1 (**C**-**D**), and F2 (**E**–**F**) generations. Males and females combined are shown in grey-scale, while separate results for the sexes are shown in blue (males) and red (females). Values are shown as mean ± SEM, along with individual data points, *n* = 17–31 individuals/treatment group/sex. Dashed line represents the threshold of preference, with values above 0.5 indicating a preference for social or novel stimuli, and values below 0.5 indicating a preference for non-social or familiar stimuli. Illustrations created with BioRender.com. Abbreviations. Bup, buprenorphine; Ctrl, control; Mtd, methadone

In the sociability phase, all groups across both generations had a preference for the social stimulus, indicated by a sociability index > 0.5 (Fig. 6C and E). Similarly, during the social novelty phase, there was a preference for the novel rat over the familiar rat, reflected by a social novelty index > 0.5 (Fig. 6D and F). Additional details regarding time spent in the different chambers are provided in Fig. S4 and S5.

#### F1 generation

There was an overall effect of treatment on the sociability index (Table S11), but the pairwise comparisons revealed no significant differences between the opioid-exposed animals and controls (Fig. 6A). There was also an overall effect of sex on the social novelty index (Table S11), where females had a higher social novelty index compared to males. No interactions between treatment and sex were found, and no differences between the treatment groups within each sex were evident (Fig. 6A-B, Table S12).

#### F2 generation

There was no main effect of treatment, either on the sociability index or the social novelty index (Table S13). We found an interaction effect between treatment and sex for the sociability index, but the pairwise comparisons revealed no difference between the treatment groups within each sex (Fig. 6C, Table S14).

## Discussion

While our understanding of the impact of prenatal exposure to MOUD on newborns and young children has expanded in recent years, significant knowledge gaps still persist regarding its long-term effects during adolescence and adulthood. Furthermore, the potential for intergenerational transmission of opioid-induced alterations has received limited attention. Across species, juvenile social play is essential for the development of social, emotional and cognitive skills (Pellis et al. 2010; Vanderschuren and Trezza 2014) and presently, there is considerable concern about how prenatal exposure to MOUD might affect this behavior. Therefore, we aimed to investigate juvenile play behavior and adult social interactions in male and female rat offspring across the F1 and F2 generations following maternal (F0) exposure to methadone or buprenorphine. We demonstrate that continuous administration of buprenorphine, but not methadone, during pregnancy results in intergenerational reduction in juvenile social play behavior, but has no discernable impact on adult social interactions.

Juvenile offspring (F1) prenatally exposed to buprenorphine exhibited reduced social play behavior, as evidenced by a reduced number of pounces and pins, which are key characteristics of social play behavior in rats (Panksepp and Beatty 1980). This reduction in play behavior was most pronounced in male offspring, although a similar, albeit non-significant, trend was also observed in females. Our results align with a recent study investigating social play behavior across three developmental stages (PND 25, 35 and 45) following perinatal morphine exposure (Harder et al. 2023). Using microinfusion minipumps, Harder and coworkers administered increasing doses of morphine (10 – 16 mg/kg/day) to pregnant rats three times daily, followed by a gradual dose reduction during the first postnatal week. Their results demonstrated reduced social play behavior, with less pouncing and pinning, when averaged across all ages in both males and females. In contrast, most previous studies report increased juvenile play behavior following prenatal morphine exposure (Buisman-Pijlman et al. 2009; Hol et al. 1996; Niesink et al. 1996). Notably, these studies have administered morphine by one daily injection to the dams during the final two weeks of gestation, resulting in fluctuating opioid concentrations. In contrast, both the present study and the study by Harder et al. used implanted pumps for continuous exposure throughout the entire gestational period. By introducing stable opioid concentrations in the blood pre-gestationally, our model strives to mimic the clinical situation where women with an OUD typically use methadone or buprenorphine before becoming pregnant. Ideally, our study should also have included exposure during the first postnatal week, corresponding to the third trimester of a human pregnancy (Clancy et al. 2007). However, due to methodological challenges associated with exposure of newborn pups, such as too low blood concentrations upon exposure through lactation, or fluctuating blood concentrations and stress associated with direct exposure of the pups, postnatal exposure was not implemented in the present study.

In accordance with our hypothesis, we also revealed reduced social play behavior in juvenile offspring of the F2 generation. Offspring of F1 parents prenatally exposed to buprenorphine exhibited reduced pouncing and pinning behaviors, mirroring the findings in their F1 predecessors, while methadone did not affect play-related behavior. Both males and females in the buprenorphine F2 group presented with reduced number of pounces and pins. However, the sample size may have restricted the ability to detect significant differences within each sex. The time spent on social exploration, i.e. sniffing or grooming the partner, was unchanged in both generations, demonstrating that buprenorphine specifically affects aspects related to more energetic social behavior, such as social play. This is similar to observations in offspring perinatally exposed to morphine, where changes in pinning, but not social grooming, were reported (Buisman-Pijlman et al. 2009). The consistency of reduced play behavior across generations highlights the potential intergenerational impact of opioid exposure, and is further supported by studies demonstrating that preconception exposure to morphine in adolescent rats leads to behavioral alterations in the offspring (Alipour et al. 2023; Azadi et al. 2019; Pachenari et al. 2019; Vassoler et al. 2016, 2017, 2022). The authors of these studies suggested that epigenetic mechanisms induced by adolescent male or female morphine exposure may be responsible for the intergenerational behavioral changes, with maternal inheritance occurring through affected oocytes, and paternal inheritance through affected sperm cells. However, there is still limited research on intergenerational effects of prenatal opioid exposure. A recent study worth noting demonstrated that prenatal exposure to oxycodone resulted in altered sociability in adult F2 rat offspring (Odegaard et al. 2020).

The reduced social play behavior in both the F1 and F2 generations after maternal (F0) buprenorphine-exposure is concerning, given the crucial role of juvenile play in the development of social, emotional and cognitive skills (Baarendse et al. 2013; Van den Berg et al. 1999). Juvenile play has been shown to facilitate the development of inhibitory prefrontal cortical synapses, which are essential for cognitive and executive functions (Bijlsma et al. 2022; Baarendse et al. 2013) and several preclinical studies have linked reduced play during the juvenile period to a number of negative outcomes in adulthood, such as increased aggression, heightened anxiety, decreased sexual behavior, and altered social interactions (Lukkes et al. 2009; Marquardt et al. 2023; Vanderschuren and Trezza 2014). Our previous studies align with these impacts, demonstrating that the opioid exposure paradigm used in the present study results in increased anxiety-like behavior (Nyberg et al. 2024) and impaired cognitive functioning (Kongstorp et al. 2020) in F1 offspring. The mechanisms by which prenatal buprenorphine exposure influences social play behavior remain unclear. However, studies suggest that prenatal exposure to opioids can modify the endogenous opioid system in the offspring by affecting opioid receptor binding and receptor densities in the brain (for review, see: Byrnes and Vassoler 2018). Since opioid neurotransmission plays a crucial role in the motivational and rewarding properties of social play behavior in rats, such alterations may lead to reduced motivation to engage in play and/or diminished reward from the play activities (Achterberg et al. 2019). Furthermore, prenatal exposure to buprenorphine has been linked to changes in synaptic signaling (Boggess et al. 2021; Niebergall et al. 2024; Simmons et al. 2023), which could lead to behavioral changes. Contrary to buprenorphine, maternal methadone exposure did not alter social play behavior in either the F1 or the F2 offspring. This discrepancy may be attributed to the pharmacological differences between methadone and buprenorphine, including their distinct receptor interactions and potential biased agonism at the µ-opioid receptor (Farid et al. 2008; Garrido and Trocóniz 1999).

The present study revealed sex-dependent effects in both generations. Buprenorphine exposure resulted in pronounced effects on social play behavior in males of the F1 generation. In the F2 generation, male offspring of methadone-exposed parents spent significantly less time on social exploration compared to controls, indicating a reduced interest in social interactions, while no effect was observed for females. Sex-dependent effects following prenatal exposure to opioids have also been shown in other preclinical studies, confirming more negative effects in males compared to females (Fleites et al. 2022; Smith et al. 2022). These findings align with clinical studies suggesting that boys appear to be more susceptible to long-term effects of prenatal opioid exposure compared to girls (Nygaard et al. 2015; Skumlien et al. 2020), and highlights the importance of including both sexes when performing preclinical studies of prenatal opioid exposure.

During social play, animals communicate by emitting USVs, where 50-kHz vocalizations are thought to indicate a positive affective state (Burgdorf et al. 2008; Burke et al. 2018). The significance of USVs in play behavior is demonstrated by studies showing reduced play in devocalized pairs (Himmler et al. 2014). Given this association between play and USV, we anticipated alterations in vocalizations to coincide with the reduced social play behavior observed in buprenorphine-exposed offspring, and performed both quantitative and qualitative analyses of USVs. However, our findings revealed no significant changes in the total number of USVs in either generation of buprenorphine-exposed animals. This suggests that the release of USVs and social play in juvenile rats may rely on different neurobehavioral mechanisms, as also demonstrated by others (Manduca et al. 2014). Although minor variations were observed in certain USV subtypes in opioid-exposed offspring, no strong correlations were established between USV subtypes and the specific play behaviors pouncing and pinning (Fig. S6). However, we noticed that USV emissions showed different associations with play behaviors compared to exploratory behaviors. Specifically, there was a tendency for a positive correlation with play and a negative correlation with exploration (Fig. S6).

Despite reduced play behavior in both F1 and F2 offspring in the buprenorphine group, we found no effect on adult social interactions when assessed in the three-chamber social interaction test. Specifically, no differences were found in the preference for the social stimulus in the sociability phase, or the novel stimulus in the social novelty phase, indicating preserved social motivation independent of treatment. The unchanged adult social interaction observed in the F1 generation aligns with the findings in a recent study, where mice perinatally exposed to buprenorphine displayed normal adult social behavior in a free-interaction setting (Smith et al. 2023). In contrast, Chen et al. reported that prenatal exposure to either methadone or buprenorphine was associated with reduced social interactions in adults (Chen et al. 2015). Odegaard et al. reported that perinatal oxycodone exposure led to a reduction in social novelty preference and overall social preference in the F2 animals, assessed with the three-chamber social interaction test, although the F1 generation remained unaffected (Odegaard et al. 2020). This discrepancy highlights that various opioids can affect social behavior differently across generations and underscores the importance of developing clinically relevant animal models.

In the present work, half of the pups in each F1 litter were separated from the dam daily from PND 2 to PND 12. We found no effect of this separation on the behavioral outcomes measured in the F1 generation in any of the treatment groups. This finding contrasts with previous studies showing that maternal separation can affect both juvenile play behavior (Kentrop et al. 2018; Veenema and Neumann 2009) and adult social interactions (Mavrenkova et al. 2023; McClafferty et al. 2024; Mejía-Chávez et al. 2021). The variations in impact of maternal separation may be attributed to the choice of separation protocol. We used a separation protocol intended to reflect reduced maternal care rather than total neglect. In retrospect, it appears that this approach, where offspring were separated in groups, was too mild to produce a negative effect, which was also suggested in our recent publication where we investigated anxiety-like behavior (Nyberg et al. 2024).

It is well-established that the use of methadone or buprenorphine during pregnancy is associated with adverse short-term outcomes in the newborns, such as preterm birth, lower birth weight and smaller head circumference (Lemon et al. 2018; Welle-Strand et al. 2013). In a recent study, we confirmed negative birth effects in rats after prenatal exposure to MOUD. Notably, decreased litter size and reduced body weight were seen (Nyberg et al. 2024), in line with other preclinical studies (Grecco et al. 2021; Lum et al. 2021; Robinson and Wallace 2001). Interestingly, no significant differences in litter size or birth weight were found in the F2 generation. However, we did observe a reduction in body weight among adult males in the buprenorphine group, potentially indicating effects on long-term physical growth. To our knowledge, no previous studies have reported short-term outcomes for the F2 generation following exposure to methadone or buprenorphine.

In the present study, we have compared the effect of maternal (F0) MOUD exposure on social behavior across two consecutive generations. It is important to emphasize that the exposure regimens of the F1 and F2 generations are not directly comparable. The F1 generation was exposed to opioids through maternal exposure during pregnancy, after mating with a drug-naïve male, ensuring direct exposure of the fetus through the maternal lineage. In contrast, the F2 generation was bred by mating an F1 male and an F1 female, both being exposed to the same substance *in utero*. This introduces the possibility that the effects in the F2 generation could stem from two distinct sources: i) exposure at the germ line stage, affecting the germ cells in male and female F1 fetuses – destined to produce the F2 generation, or ii) epigenetic modifications passed down from the maternal and/or paternal lineage. Indeed, several studies attribute changes observed in the F2 generation to the paternal lineage (Choi et al. 2016; Luo et al. 2023; Zappala et al. 2023), including studies of morphine exposure of adolescent males (Vassoler et al. 2020; Zeid et al. 2023). Future studies are needed to fully understand the underlying mechanisms of inherited effects of prenatal opioid exposure. Extending investigations to the F3 generation, which represents the first generation without direct exposure, could illuminate whether the observed opioid-induced behavioral alterations in the present study represent true transgenerational effects.

## Conclusion

Our study is the first to demonstrate intergenerational effects of exposure to MOUD during pregnancy. Specifically, we discovered that maternal exposure to buprenorphine resulted in reduced social play behavior in both F1 and F2 offspring, without altering USV. In the F1 generation, males appeared more susceptible to the impacts of prenatal buprenorphine exposure compared to females. The altered juvenile play behavior did not affect social interactions in adulthood. Interestingly, no effect was found for methadone, either regarding juvenile play behavior or adult social interactions in either generations. Future studies should aim to elucidate the mechanisms behind the intergenerational effects, with a particular focus on epigenetic modifications.

## Supplementary Information

Below is the link to the electronic supplementary material.

Supplementary file1 (DOCX 4.81 MB)

## Abbreviations

Bup: Buprenorphine
LMM: Linear mixed model
MS: Maternal separation
Mtd: Methadone
MOUD: Medications for opioid use disorder
OUD: Opioid use disorder
PND: Postnatal day
USV: Ultrasonic vocalization
