# Supplementary material for: Maternal exposure to buprenorphine, but not methadone, during pregnancy reduces social play behavior across two generations of offspring
Source: Psychopharmacology (Berl). 2024 Dec 5;242(3):663–80. doi: 10.1007/s00213-024-06718-2 (PMC11861248; doi:10.1007/s00213-024-06718-2)
Supplement: Supplementary file 1 — Supplementary file1 (DOCX 4.81 MB) [file 213_2024_6718_MOESM1_ESM.docx]

**Supplementary Information (SI)**

**Journal name: Psychopharmacology**

**Title: Maternal exposure to buprenorphine, but not methadone, during pregnancy reduces social play behavior across two generations of offspring**

Henriette Nyberg^a,b^, Inger Lise Bogen^a,b^, Egil Nygaard^c^, Marijke Achterberg^d^ and Jannike Mørch Andersen^a,b^

1. Department of Forensic Sciences, Section of Forensic Research, Oslo University Hospital, Oslo, Norway
2. Department of Pharmacy, Faculty of Mathematics and Natural Sciences, University of Oslo, Oslo, Norway
3. Department of Psychology, PROMENTA, Faculty of Social Sciences, University of Oslo, Oslo, Norway
4. Department of Population Health Sciences, Behavioral Neuroscience group, Faculty of Veterinary Medicine, University of Utrecht, The Netherlands

**Manuscript correspondence:**

Henriette Nyberg
Section of Forensic Research, Department of Forensic Sciences, Oslo University Hospital
Address: PO Box 4950 Nydalen, Oslo, Norway
Email: hennyb@ous-hf.no

| **Table S1** Number of pairs and intraclass correlation coefficients for ‘batch’ for social play and ultrasonic vocalizations across the F1 and F2 generations. | | | |
| --- | --- | --- | --- |
|  |  | **n (pairs)** | **ICC** |
| **Social play F1** | Pouncing | 73 | 0.33 |
|  | Pinning | 73 | 0.20 |
|  | Social exploration | 73 | 0.31 |
|  | Nonsocial exploration | 73 | 0.07 |
| **Social play F2** | Pouncing | 84 | 0.50 |
|  | Pinning | 84 | 0.35 |
|  | Social exploration | 84 | 0.20 |
|  | Nonsocial exploration | 84 | 0.48 |
| **USV F1** | Total calls | 36 | 0.12 |
|  | Flat | 36 | R. |
|  | Step | 36 | 0.21 |
|  | Short | 36 | 0.04 |
|  | Trill | 36 | 0.34 |
|  | Other | 36 | R. |
| **USV F2** | Total calls | 44 | 0.02 |
|  | Flat | 44 | 0.11 |
|  | Step | 44 | 0.31 |
|  | Short | 44 | R. |
|  | Trill | 44 | R. |
|  | Other | 44 | 0.11 |
| **Notes.** ICC-values obtained from a null model without fixed effects, batch included as random intercept only.  **Abbreviations.** ICC, intraclass correlation coefficient; USV, ultrasonic vocalization; R, redundant | | | |

| **Table S2** Number of observations and intraclass correlation coefficients for ‘litter’ and ‘batch’, as well as ‘litter + batch’ for the three-chamber social interaction test across the F1 and F2 generations. | | | | | |
| --- | --- | --- | --- | --- | --- |
|  |  |  | **ICC** | | |
|  |  | **n (individuals)** | **Litter** | **Batch** | **Litter + batch** |
| **Three-chamber social interaction F1** | Social stimulus | 168 | 0.08 | R. | 0.08 |
|  | Nonsocial stimulus | 168 | 0.13 | 0.15 | 0.17 |
|  | Novel stimulus | 168 | R. | R. | R. |
|  | Familiar stimulus | 168 | 0.07 | 0.15 | 0.15 |
|  | Sociability index | 168 | 0.10 | 0.07 | 0.08 |
|  | Social novelty index | 168 | R. | 0.05 | R. |
| **Three-chamber social interaction F2** | Social stimulus | 120 | R. | 0.05 | 0.05 |
|  | Nonsocial stimulus | 120 | R. | 0.03 | 0.03 |
|  | Novel stimulus | 120 | R. | 0.15 | 0.17 |
|  | Familiar stimulus | 120 | R. | R. | R. |
|  | Sociability index | 120 | R. | R. | R. |
|  | Social novelty index | 120 | R. | 0.02 | R. |
| **Notes.** ICC-values obtained from null model without fixed effects, litter and/or batch included as random intercepts only. **Abbreviations.** ICC, intraclass correlation coefficient; R, redundant | | | | | |

| **Table S3** Effects of maternal exposure (F0) to methadone (10 mg/kg/day) or buprenorphine (1 mg/kg/day) on social play behavior in the F1 generation. | | | | |
| --- | --- | --- | --- | --- |
|  | **Df1** | **Df2** | **F-value** | **p-value** |
| **Pouncing** |  |  |  |  |
| Treatment | 2 | 64 | **4.417** | **0.016** |
| Sex | 1 | 64 | **11.532** | **0.001** |
| Maternal separation (MS) | 1 | 64 | 0.165 | 0.686 |
| Treatment*Sex | 2 | 64 | **3.084** | **0.053** |
| Treatment*MS | 2 | 64 | 1.936 | 0.153 |
|  |  |  |  |  |
| **Pinning** |  |  |  |  |
| Treatment | 2 | 64 | **6.860** | **0.002** |
| Sex | 1 | 64 | **9.172** | **0.004** |
| Maternal separation (MS) | 1 | 64 | 0.018 | 0.893 |
| Treatment*Sex | 2 | 64 | 2.397 | 0.099 |
| Treatment*MS | 2 | 64 | 1.682 | 0.194 |
|  |  |  |  |  |
| **Social exploration** |  |  |  |  |
| Treatment | 2 | 62.5 | 2.255 | 0.113 |
| Sex | 1 | 62.05 | 0.004 | 0.949 |
| Maternal separation (MS) | 1 | 62.01 | 0.406 | 0.526 |
| Treatment*Sex | 2 | 62.02 | 0.483 | 0.620 |
| Treatment*MS | 2 | 62.00 | 0.092 | 0.912 |
|  |  |  |  |  |
| **Non-social exploration** |  |  |  |  |
| Treatment | 2 | 64 | 1.045 | 0.358 |
| Sex | 1 | 64 | **9.269** | **0.003** |
| Maternal separation (MS) | 1 | 64 | 0.332 | 0.565 |
| Treatment*Sex | 2 | 64 | 1.923 | 0.155 |
| Treatment*MS | 2 | 64 | 1.553 | 0.220 |
| **Notes.** Statistical analyses were performed with a LMM with treatment, sex and maternal separation, as well as the treatment*sex and treatment*maternal separation interactions as fixed factors. Batch was included as a random factor when the ICC > 0.1. **Abbreviations.** Df, degrees of freedom; ICC, intraclass correlation coefficient; LMM, linear mixed model. | | | | |

| **Table S4** Effects of maternal exposure (F0) to methadone (10 mg/kg/day) or buprenorphine (1 mg/kg/day) on social play behavior in the F2 generation. | | | | |
| --- | --- | --- | --- | --- |
|  | **Df1** | **Df2** | **F-value** | **p-value** |
| **Pouncing** |  |  |  |  |
| Treatment | 2 | 76.02 | **5.875** | **0.004** |
| Sex | 1 | 76.13 | 2.495 | 0.118 |
| Treatment*Sex | 2 | 76.02 | 0.155 | 0.857 |
|  |  |  |  |  |
| **Pinning** |  |  |  |  |
| Treatment | 2 | 76.03 | **4.480** | **0.015** |
| Sex | 1 | 76.23 | 0.753 | 0.388 |
| Treatment*Sex | 2 | 76.02 | 0.221 | 0.802 |
|  |  |  |  |  |
| **Social exploration** |  |  |  |  |
| Treatment | 2 | 75.09 | **4.596** | **0.013** |
| Sex | 1 | 76.52 | 0.285 | 0.595 |
| Treatment*Sex | 2 | 76.05 | 0.643 | 0.529 |
|  |  |  |  |  |
| **Non-social exploration** |  |  |  |  |
| Treatment | 2 | 76.01 | **4.155** | **0.019** |
| Sex | 1 | 76.10 | **5.199** | **0.025** |
| Treatment*Sex | 2 | 76.01 | 0.225 | 0.799 |
| **Notes.** Statistical analyses were performed with a LMM with treatment and sex, as well as the treatment*sex interaction as fixed factors. Batch was included as a random factor when the ICC > 0.1. **Abbreviations.** Df, degrees of freedom; ICC, intraclass correlation coefficient; LMM, linear mixed model. | | | | |

| **Table S5** Social play behavior in the F1 generation. Pairwise comparisons of treatment versus control within each sex. | | | | | | |
| --- | --- | --- | --- | --- | --- | --- |
|  |  | **Estimate** | **SE** | **Df** | **t-value** | **p-value** |
|  | **Pouncing** |  |  |  |  |  |
| ♂ | Methadone – Control | 5.37 | 7.68 | 64 | 0.699 | 0.7017 |
|  | Buprenorphine – Control | -24.06 | 8.83 | 64 | 2.725 | **0.0160** |
|  |  |  |  |  |  |  |
| ♀ | Methadone – Control | -12.00 | 8.37 | 64 | -1.434 | 0.2717 |
|  | Buprenorphine – Control | -10.67 | 8.17 | 64 | -1.306 | 0.3333 |
|  |  |  |  |  |  |  |
|  | **Pinning** |  |  |  |  |  |
| ♂ | Methadone – Control | 1.14 | 4.32 | 64 | 0.264 | 0.9384 |
|  | Buprenorphine – Control | -17.00 | 4.97 | 64 | -3.422 | **0.0021** |
|  |  |  |  |  |  |  |
| ♀ | Methadone – Control | -3.89 | 4.71 | 64 | -0.827 | 0.6192 |
|  | Buprenorphine – Control | -6.78 | 4.60 | 64 | -1.467 | 0.2535 |
|  |  |  |  |  |  |  |
|  | **Social exploration** |  |  |  |  |  |
| ♂ | Methadone – Control | 28.4 | 20.9 | 62.4 | 1.358 | 0.3077 |
|  | Buprenorphine – Control | 46.3 | 25.0 | 63.1 | 1.853 | 0.1257 |
|  |  |  |  |  |  |  |
| ♀ | Methadone – Control | 24.8 | 22.6 | 62.2 | 1.097 | 0.4490 |
|  | Buprenorphine – Control | 46.3 | 25.0 | 63.3 | 0.693 | 0.7056 |
|  |  |  |  |  |  |  |
|  | **Non-social exploration** |  |  |  |  |  |
| ♂ | Methadone – Control | -59.28 | 25.1 | 64 | -2.365 | **0.0401** |
|  | Buprenorphine – Control | -17.16 | 28.8 | 64 | -0.595 | 0.7668 |
|  |  |  |  |  |  |  |
| ♀ | Methadone – Control | 6.56 | 27.3 | 64 | 0.240 | 0.9474 |
|  | Buprenorphine – Control | -16.50 | 26.7 | 64 | -0.618 | 0.7524 |
| **Notes.** Linear mixed model with treatment, sex, treatment*sex, and treatment*maternal separation as fixed factors. Batch included as a random intercept when the ICC > 0.1. Dunnett correction for multiple comparisons. **Abbreviations.** Df, degrees of freedom; ICC, intraclass correlation coefficient; SE, standard error. | | | | | | |

| **Table S6** Social play behavior in the F2 generation. Pairwise comparisons of treatment versus control within each sex. | | | | | | |
| --- | --- | --- | --- | --- | --- | --- |
|  |  | **Estimate** | **SE** | **Df** | **t-value** | **p-value** |
|  | **Pouncing** |  |  |  |  |  |
| ♂ | Methadone – Control | 0.805 | 8.67 | 76.0 | 0.093 | 0.9893 |
|  | Buprenorphine – Control | -19.945 | 9.05 | 76.7 | -2.204 | 0.0577 |
|  |  |  |  |  |  |  |
| ♀ | Methadone – Control | 7.288 | 8.93 | 76.0 | 0.816 | 0.6257 |
|  | Buprenorphine – Control | -18.912 | 9.66 | 77.2 | -1.958 | 0.0999 |
|  |  |  |  |  |  |  |
|  | **Pinning** |  |  |  |  |  |
| ♂ | Methadone – Control | 0.161 | 4.76 | 76.0 | 0.034 | 0.9980 |
|  | Buprenorphine – Control | -8.628 | 4.98 | 77.1 | -1.734 | 0.1576 |
|  |  |  |  |  |  |  |
| ♀ | Methadone – Control | 2.431 | 4.90 | 76.0 | 0.496 | 0.8252 |
|  | Buprenorphine – Control | -10.944 | 5.32 | 77.8 | -2.056 | 0.0805 |
|  |  |  |  |  |  |  |
|  | **Social exploration** |  |  |  |  |  |
| ♂ | Methadone – Control | -50.34 | 19.9 | 76.1 | -2.536 | **0.0255** |
|  | Buprenorphine – Control | 7.22 | 20.9 | 77.9 | 0.346 | 0.9030 |
|  |  |  |  |  |  |  |
| ♀ | Methadone – Control | -21.32 | 20.4 | 76.0 | -1.043 | 0.4813 |
|  | Buprenorphine – Control | 9.05 | 22.4 | 77.1 | 0.404 | 0.8749 |
|  |  |  |  |  |  |  |
|  | **Non-social exploration** |  |  |  |  |  |
| ♂ | Methadone – Control | 33.2 | 22.3 | 76.0 | 1.486 | 0.2477 |
|  | Buprenorphine – Control | 51.6 | 23.3 | 76.5 | 2.213 | 0.0564 |
|  |  |  |  |  |  |  |
| ♀ | Methadone – Control | 14.5 | 23.0 | 76.0 | 0.632 | 0.7438 |
|  | Buprenorphine – Control | 51.8 | 24.9 | 77.0 | 2.083 | 0.0760 |

**Notes.** Linear mixed model with treatment, sex, and treatment*sex as fixed factors. Batch included as a random intercept when the ICC > 0.1. Dunnett correction for multiple comparisons.
**Abbreviations.** Df, degrees of freedom; ICC, intraclass correlation coefficient; SE, standard error.

| **Table S7** Effects of maternal exposure (F0) to methadone (10 mg/kg/day) or buprenorphine (1 mg/kg/day) on ultrasonic vocalizations in the F1 generation. | | | | |
| --- | --- | --- | --- | --- |
|  | **Df1** | **Df2** | **F-value** | **p-value** |
| **Total calls** |  |  |  |  |
| Treatment | 2 | 27 | 0.150 | 0.8611 |
| Sex | 1 | 27 | 4.154 | **0.0514** |
| Maternal separation (MS) | 1 | 27 | 0.539 | 0.4691 |
| Treatment*Sex | 2 | 27 | 3.305 | **0.0520** |
| Treatment*MS | 2 | 27 | 0.306 | 0.7392 |
|  |  |  |  |  |
| **Flat** |  |  |  |  |
| Treatment | 2 | 27 | 0.303 | 0.7410 |
| Sex | 1 | 27 | 0.404 | 0.5302 |
| Maternal separation (MS) | 1 | 27 | 0.068 | 0.7964 |
| Treatment*Sex | 2 | 27 | 1.074 | 0.3558 |
| Treatment*MS | 2 | 27 | 0.773 | 0.4717 |
|  |  |  |  |  |
| **Step** |  |  |  |  |
| Treatment | 2 | 26.00 | 0.449 | 0.6430 |
| Sex | 1 | 26.00 | 0.133 | 0.7179 |
| Maternal separation (MS) | 1 | 26.32 | 0.094 | 0.7612 |
| Treatment*Sex | 2 | 26.01 | 0.089 | 0.9147 |
| Treatment*MS | 2 | 26.01 | 0.673 | 0.5186 |
|  |  |  |  |  |
| **Short** |  |  |  |  |
| Treatment | 2 | 27 | 0.321 | 0.7281 |
| Sex | 1 | 27 | 1.096 | 0.3045 |
| Maternal separation (MS) | 1 | 27 | 0.007 | 0.9323 |
| Treatment*Sex | 2 | 27 | 1.889 | 0.1707 |
| Treatment*MS | 2 | 27 | 1.152 | 0.3311 |
|  |  |  |  |  |
| **Trill** |  |  |  |  |
| Treatment | 2 | 27 | 1.560 | 0.2284 |
| Sex | 1 | 27 | 4.807 | **0.0371** |
| Maternal separation (MS) | 1 | 27 | 0.123 | 0.7289 |
| Treatment*Sex | 2 | 27 | 3.272 | **0.0534** |
| Treatment*MS | 2 | 27 | 0.255 | 0.7771 |
|  |  |  |  |  |
| **Other** |  |  |  |  |
| Treatment | 2 | 27 | 0.292 | 0.7492 |
| Sex | 1 | 27 | 2.782 | 0.1069 |
| Maternal separation (MS) | 1 | 27 | 0.989 | 0.3288 |
| Treatment*Sex | 2 | 27 | 2.664 | 0.0879 |
| Treatment*MS | 2 | 27 | 0.255 | 0.7766 |
| **Notes.** Linear mixed model with treatment, sex, treatment*sex, and treatment*maternal separation as fixed factors. Batch included as a random intercept when the ICC > 0.1. **Abbreviations.** Df, degrees of freedom; ICC, intraclass correlation coefficient. | | | | |

| **Table S8** Ultrasonic vocalizations in the F1 generation. Pairwise comparisons of treatment versus control within each sex. | | | | | | |
| --- | --- | --- | --- | --- | --- | --- |
|  |  | **Estimate** | **SE** | **Df** | **t-value** | **p-value** |
|  | **Total calls** |  |  |  |  |  |
| ♂ | Methadone – Control | -144.4 | 92.6 | 27 | -1.560 | 0.2294 |
|  | Buprenorphine – Control | -174.3 | 96.3 | 27 | -1.810 | 0.1475 |
|  |  |  |  |  |  |  |
| ♀ | Methadone – Control | 76.2 | 96.9 | 27 | 0.786 | 0.6491 |
|  | Buprenorphine – Control | 169.5 | 95.5 | 27 | 1.774 | 0.1576 |
|  |  |  |  |  |  |  |
|  | **Flat** |  |  |  |  |  |
| ♂ | Methadone – Control | 1.54 | 2.70 | 27 | 0.570 | 0.7837 |
|  | Buprenorphine – Control | 4.36 | 2.81 | 27 | 1.550 | 0.2333 |
|  |  |  |  |  |  |  |
| ♀ | Methadone – Control | -1.17 | 2.83 | 27 | -0.412 | 0.8717 |
|  | Buprenorphine – Control | -1.48 | 2.79 | 27 | -0.530 | 0.8072 |
|  |  |  |  |  |  |  |
|  | **Step** |  |  |  |  |  |
| ♂ | Methadone – Control | -6.042 | 7.14 | 26 | -0.846 | 0.6111 |
|  | Buprenorphine – Control | -0.547 | 8.44 | 27 | -0.065 | 0.9942 |
|  |  |  |  |  |  |  |
| ♀ | Methadone – Control | -2.300 | 7.47 | 26 | -0.308 | 0.9209 |
|  | Buprenorphine – Control | 3.269 | 8.54 | 27 | 0.383 | 0.8864 |
|  |  |  |  |  |  |  |
|  | **Short** |  |  |  |  |  |
| ♂ | Methadone – Control | -25.9 | 14.1 | 27 | -1.843 | 0.1386 |
|  | Buprenorphine – Control | -18.6 | 14.6 | 27 | -1.271 | 0.3598 |
|  |  |  |  |  |  |  |
| ♀ | Methadone – Control | 9.7 | 14.7 | 27 | 0.659 | 0.7299 |
|  | Buprenorphine – Control | 13.8 | 14.5 | 27 | 0.950 | 0.5452 |
|  |  |  |  |  |  |  |
|  | **Trill** |  |  |  |  |  |
| ♂ | Methadone – Control | -36.65 | 36.7 | 27 | -1.000 | 0.5142 |
|  | Buprenorphine – Control | -34.65 | 38.1 | 27 | -0.909 | 0.5712 |
|  |  |  |  |  |  |  |
| ♀ | Methadone – Control | 7.96 | 38.4 | 27 | 0.207 | 0.9591 |
|  | Buprenorphine – Control | 101.63 | 37.8 | 27 | 2.687 | **0.0233** |
|  |  |  |  |  |  |  |
|  | **Other** |  |  |  |  |  |
| ♂ | Methadone – Control | -77.7 | 55.7 | 27 | -1.394 | 0.2994 |
|  | Buprenorphine – Control | -119.1 | 58.0 | 27 | -2.054 | 0.0922 |
|  |  |  |  |  |  |  |
| ♀ | Methadone – Control | 62.1 | 58.4 | 27 | 1.064 | 0.4753 |
|  | Buprenorphine – Control | 58.5 | 57.5 | 27 | 1.018 | 0.5030 |
|  |  |  |  |  |  |  |

**Notes.** Linear mixed model with treatment, sex, treatment*sex, and treatment*maternal separation as fixed factors. Batch included as a random intercept when the ICC > 0.1. Dunnett correction for multiple comparisons
**Abbreviations.** Df, degrees of freedom; ICC, intraclass correlation coefficient; SE, standard error.

| **Table S9** Effects of maternal exposure (F0) to methadone (10 mg/kg/day) or buprenorphine (1 mg/kg/day) on ultrasonic vocalizations in the F2 generation. | | | | |
| --- | --- | --- | --- | --- |
|  | **Df1** | **Df2** | **F-value** | **p-value** |
| **Total calls** |  |  |  |  |
| Treatment | 2 | 38 | 1.294 | 0.2861 |
| Sex | 1 | 38 | 2.361 | 0.1327 |
| Treatment*Sex | 2 | 38 | 2.242 | 0.1202 |
|  |  |  |  |  |
| **Flat** |  |  |  |  |
| Treatment | 2 | 36.07 | 0.174 | 0.8407 |
| Sex | 1 | 32.22 | 0.656 | 0.4239 |
| Treatment*Sex | 2 | 37.20 | 0.177 | 0.8384 |
|  |  |  |  |  |
| **Step** |  |  |  |  |
| Treatment | 2 | 36.05 | 0.869 | 0.4282 |
| Sex | 1 | 38.00 | 0.378 | 0.5424 |
| Treatment*Sex | 2 | 36.40 | 0.566 | 0.5724 |
|  |  |  |  |  |
| **Short** |  |  |  |  |
| Treatment | 2 | 38 | 3.445 | **0.0422** |
| Sex | 1 | 38 | 4.428 | **0.0420** |
| Treatment*Sex | 2 | 38 | 6.074 | **0.0051** |
|  |  |  |  |  |
| **Trill** |  |  |  |  |
| Treatment | 2 | 38 | 3.740 | **0.0329** |
| Sex | 1 | 38 | 6.520 | **0.0148** |
| Treatment*Sex | 2 | 38 | 0.542 | 0.5862 |
|  |  |  |  |  |
| **Other** |  |  |  |  |
| Treatment | 2 | 36.07 | 0.066 | 0.9360 |
| Sex | 1 | 37.82 | 1.817 | 0.1857 |
| Treatment*Sex | 2 | 36.54 | 4.696 | **0.0153** |
| **Notes.** Linear mixed model with treatment, sex, and treatment*sex as fixed factors. Batch included as a random intercept when the ICC > 0.1. **Abbreviations.** Df, degrees of freedom; ICC, intraclass correlation coefficient. | | | | |

| **Table S10** Ultrasonic vocalizations in the F2 generation. Pairwise comparisons of treatment versus control within each sex. | | | | | | |
| --- | --- | --- | --- | --- | --- | --- |
|  |  | **Estimate** | **SE** | **Df** | **t-value** | **p-value** |
|  | **Total calls** |  |  |  |  |  |
| ♂ | Methadone – Control | 101.2 | 78.3 | 38 | 1.293 | 0.3442 |
|  | Buprenorphine – Control | 196.9 | 95.9 | 38 | 2.053 | 0.0874 |
|  |  |  |  |  |  |  |
| ♀ | Methadone – Control | 66.2 | 78.3 | 38 | 0.846 | 0.6089 |
|  | Buprenorphine – Control | -52.5 | 78.3 | 38 | -0.670 | 0.7212 |
|  |  |  |  |  |  |  |
|  | **Flat** |  |  |  |  |  |
| ♂ | Methadone – Control | -0.542 | 2.56 | 36.3 | -0.212 | 0.9576 |
|  | Buprenorphine – Control | 1.664 | 3.52 | 37.8 | 0.473 | 0.8388 |
|  |  |  |  |  |  |  |
| ♀ | Methadone – Control | -1.165 | 2.62 | 37.1 | -0.444 | 0.8544 |
|  | Buprenorphine – Control | -0.799 | 2.85 | 37.7 | -0.281 | 0.9318 |
|  |  |  |  |  |  |  |
|  | **Step** |  |  |  |  |  |
| ♂ | Methadone – Control | -12.67 | 9.03 | 36.1 | -1.403 | 0.2911 |
|  | Buprenorphine – Control | 2.88 | 12.04 | 37.2 | 0.239 | 0.9480 |
|  |  |  |  |  |  |  |
| ♀ | Methadone – Control | -2.65 | 9.19 | 36.4 | -0.288 | 0.9288 |
|  | Buprenorphine – Control | -1.62 | 9.87 | 37.4 | -0.164 | 0.9724 |
|  |  |  |  |  |  |  |
|  | **Short** |  |  |  |  |  |
| ♂ | Methadone – Control | 14.88 | 11.2 | 38 | 1.327 | 0.3270 |
|  | Buprenorphine – Control | 53.25 | 13.7 | 38 | 3.879 | **0.0008** |
|  |  |  |  |  |  |  |
| ♀ | Methadone – Control | 2.62 | 11.2 | 38 | 0.234 | 0.9498 |
|  | Buprenorphine – Control | -6.75 | 11.2 | 38 | -0.602 | 0.7635 |
|  |  |  |  |  |  |  |
|  | **Trill** |  |  |  |  |  |
| ♂ | Methadone – Control | 95.4 | 39.7 | 38 | 2.403 | **0.0404** |
|  | Buprenorphine – Control | 86.2 | 48.6 | 38 | 1.774 | 0.1523 |
|  |  |  |  |  |  |  |
| ♀ | Methadone – Control | 53.9 | 39.7 | 38 | 1.357 | 0.3122 |
|  | Buprenorphine – Control | 24.4 | 39.7 | 38 | 0.614 | 0.7562 |
|  |  |  |  |  |  |  |
|  | **Other** |  |  |  |  |  |
| ♂ | Methadone – Control | 12.81 | 35.9 | 36.1 | 0.357 | 0.8986 |
|  | Buprenorphine – Control | 95.79 | 48.1 | 37.5 | 1.993 | 0.0991 |
|  |  |  |  |  |  |  |
| ♀ | Methadone – Control | -3.61 | 36.6 | 36.5 | -0.099 | 0.9882 |
|  | Buprenorphine – Control | -72.37 | 39.4 | 37.7 | -1.838 | 0.1349 |
|  |  |  |  |  |  |  |

**Notes.** Linear mixed model with treatment, sex, and treatment*sex as fixed factors. Batch included as a random intercept when the ICC > 0.1. Dunnett correction for multiple comparisons.
**Abbreviations.** Df, degrees of freedom; ICC, intraclass correlation coefficient; SE, standard error.

| **Table S11** Effects of maternal exposure (F0) to methadone (10 mg/kg/day) or buprenorphine (1 mg/kg/day) on social interactions in the F1 generation. | | | | |
| --- | --- | --- | --- | --- |
|  | **Df1** | **Df2** | **F-ratio** | **p-value** |
| **Sociability index** | | | | |
| Treatment | 2 | 159 | 4.404 | **0.0138** |
| Sex | 1 | 159 | 0.152 | 0.6967 |
| Maternal separation (MS) | 1 | 159 | 0.587 | 0.4449 |
| Treatment*Sex | 2 | 159 | 0.582 | 0.5600 |
| Treatment*MS | 2 | 159 | 2.134 | 0.1217 |
|  |  |  |  |  |
| **Social novelty index** | | | | |
| Treatment | 2 | 159 | 2.567 | 0.0792 |
| Sex | 1 | 159 | 6.685 | **0.0106** |
| Maternal separation (MS) | 1 | 159 | 0.358 | 0.5506 |
| Treatment*Sex | 2 | 159 | 0.048 | 0.9527 |
| Treatment*MS | 2 | 159 | 1.263 | 0.2856 |
| **Notes.** Linear mixed model with treatment, sex, and treatment*sex and treatment*maternal separation as fixed factors. Batch and litter included as random intercepts when the ICC > 0.1. **Abbreviations.** Df, degrees of freedom; ICC, intraclass correlation coefficient. | | | | |

| **Table S12** Three-chamber social interaction test in the F1 generation. Pairwise comparisons of treatment versus control within each sex. | | | | | | |
| --- | --- | --- | --- | --- | --- | --- |
|  |  | **Estimate** | **SE** | **Df** | **t-value** | **p-value** |
|  | **Sociability index** |  |  |  |  |  |
| ♂ | Methadone – Control | -0.0141 | 0.0283 | 159 | -0.499 | 0.8231 |
|  | Buprenorphine – Control | 0.0567 | 0.0275 | 159 | 2.063 | 0.0764 |
|  |  |  |  |  |  |  |
| ♀ | Methadone – Control | -0.0349 | 0.0284 | 159 | -1.230 | 0.3695 |
|  | Buprenorphine – Control | 0.0145 | 0.0278 | 159 | 0.521 | 0.8101 |
|  |  |  |  |  |  |  |
|  | **Social novelty index** |  |  |  |  |  |
| ♂ | Methadone – Control | -0.0393 | 0.0299 | 159 | -1.317 | 0.3239 |
|  | Buprenorphine – Control | 0.0110 | 0.0290 | 159 | 0.379 | 0.8870 |
|  |  |  |  |  |  |  |
| ♀ | Methadone – Control | -0.0402 | 0.0299 | 159 | -1.343 | 0.3105 |
|  | Buprenorphine – Control | -0.0008 | 0.0294 | 159 | -0.026 | 0.9987 |
| **Notes.** Linear mixed model with treatment, sex, treatment*sex, and treatment*maternal separation as fixed factors. Batch and litter included as random intercepts when the ICC > 0.1. Dunnett correction for multiple comparisons. **Abbreviations.** Df, degrees of freedom; ICC, intraclass correlation coefficient; SE, standard error. | | | | | | |

| **Table S13** Effects of maternal exposure (F0) to methadone (10 mg/kg/day) or buprenorphine (1 mg/kg/day) on social interactions in the F2 generation. | | | | |
| --- | --- | --- | --- | --- |
|  | **Df1** | **Df2** | **F-ratio** | **p-value** |
| **Sociability index** | | | | |
| Treatment | 2 | 114 | 0.228 | 0.7967 |
| Sex | 1 | 114 | 0.135 | 0.7137 |
| Treatment*Sex | 2 | 114 | 3.561 | **0.0316** |
|  |  |  |  |  |
| **Social novelty index** | | | | |
| Treatment | 2 | 114 | 0.073 | 0.9301 |
| Sex | 1 | 114 | 0.289 | 0.5919 |
| Treatment*Sex | 2 | 114 | 0.136 | 0.8734 |
| **Notes.** Linear mixed model with treatment, sex, and treatment*sex as fixed factors. Batch and litter included as random intercepts when the ICC > 0.1. **Abbreviations.** Df, degrees of freedom; ICC, intraclass correlation coefficient. | | | | |

| **Table S14** Three-chamber social interaction test in the F2 generation. Pairwise comparisons of treatment versus control within each sex. | | | | | | |
| --- | --- | --- | --- | --- | --- | --- |
|  |  | **Estimate** | **SE** | **Df** | **t-value** | **p-value** |
|  | **Sociability index** |  |  |  |  |  |
| ♂ | Methadone – Control | 0.03146 | 0.0337 | 114 | 0.933 | 0.5488 |
|  | Buprenorphine – Control | -0.0417 | 0.0349 | 114 | -1.193 | 0.3910 |
|  |  |  |  |  |  |  |
| ♀ | Methadone – Control | -0.0041 | 0.0315 | 114 | -0.131 | 0.9810 |
|  | Buprenorphine – Control | 0.0429 | 0.0315 | 114 | 1.362 | 0.3023 |
|  |  |  |  |  |  |  |
|  | **Social novelty index** |  |  |  |  |  |
| ♂ | Methadone – Control | -0.0155 | 0.0389 | 114 | -0.399 | 0.8771 |
|  | Buprenorphine – Control | -0.02368 | 0.0403 | 114 | -0.588 | 0.7705 |
|  |  |  |  |  |  |  |
| ♀ | Methadone – Control | 0.0057 | 0.0363 | 114 | 0.156 | 0.9746 |
|  | Buprenorphine – Control | 0.0030 | 0.0363 | 114 | 0.083 | 0.9911 |
| **Notes.** Linear mixed model with treatment, sex, treatment*sex, and treatment*maternal separation as fixed factors. Batch and litter included as random intercepts when the ICC > 0.1. Dunnett correction for multiple comparisons. **Abbreviations.** Df, degrees of freedom; ICC, intraclass correlation coefficient; SE, standard error. | | | | | | |

| 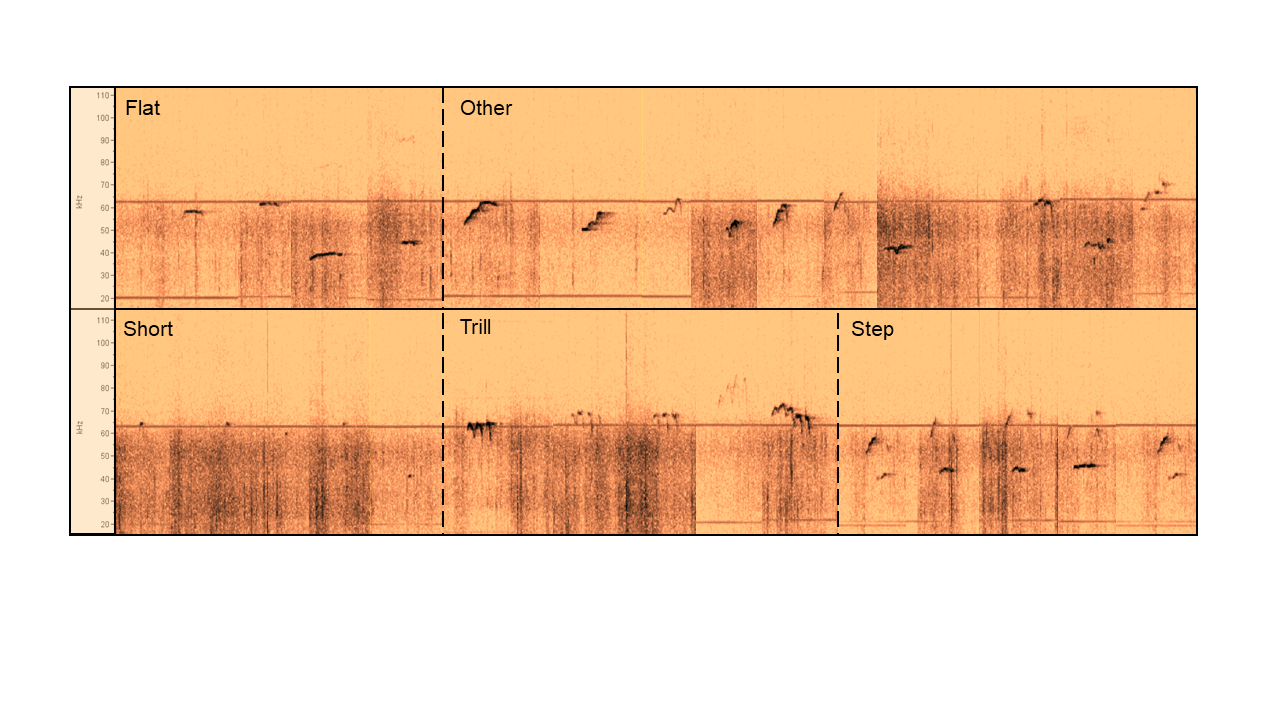 |
| --- |
| **Fig. S1** Representative examples of ultrasonic vocalization categories in the present work: flat, other, short, trill and step. |

| 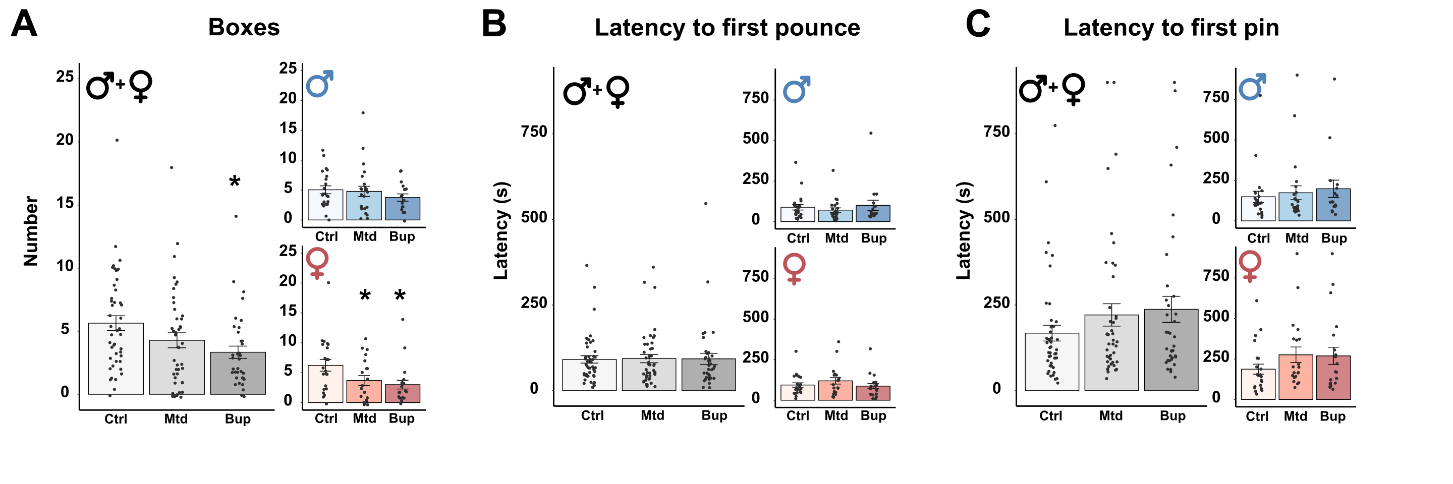 |
| --- |
| **Fig. S2** Social play behavior in the F1 generation. Number of boxes (A) and the latency to first pounce (B) and latency to first pin (C) in male and female offspring exposed to methadone (10 mg/kg/day) or buprenorphine (1 mg/kg/day) *in utero*. Males and females combined are shown in grey-scale, while separate results for the sexes are shown in blue (males) and red (females). All data are collapsed across maternal separation status. Values are shown as mean ± SEM, along with data points representing pairs of rats, n = 8 - 15 pairs/treatment group/sex. *p < 0.05, compared to control. Abbreviations. Bup, buprenorphine; Ctrl, control; Mtd, methadone |

| 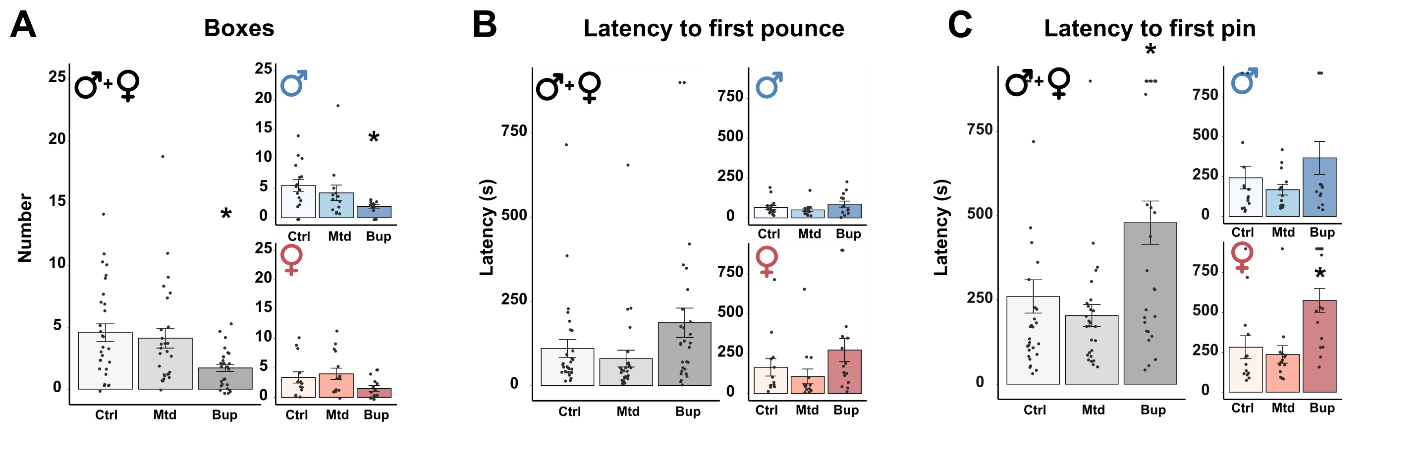 |
| --- |
| **Fig. S3** Social play behavior in the F2 generation. Number of boxes (A) and the latency to first pounce (B) and latency to first pin (C) in male and female offspring born to parents exposed to methadone (10 mg/kg/day) or buprenorphine (1 mg/kg/day) *in utero*. Males and females combined are shown in grey-scale, while separate results for the sexes are shown in blue (males) and red (females). Values are shown as mean ± SEM, along with data points representing pairs of rats, n = 13 - 16 pairs/treatment group/sex. *p < 0.05, compared to control. Abbreviations. Bup, buprenorphine; Ctrl, control; Mtd, methadone |

| 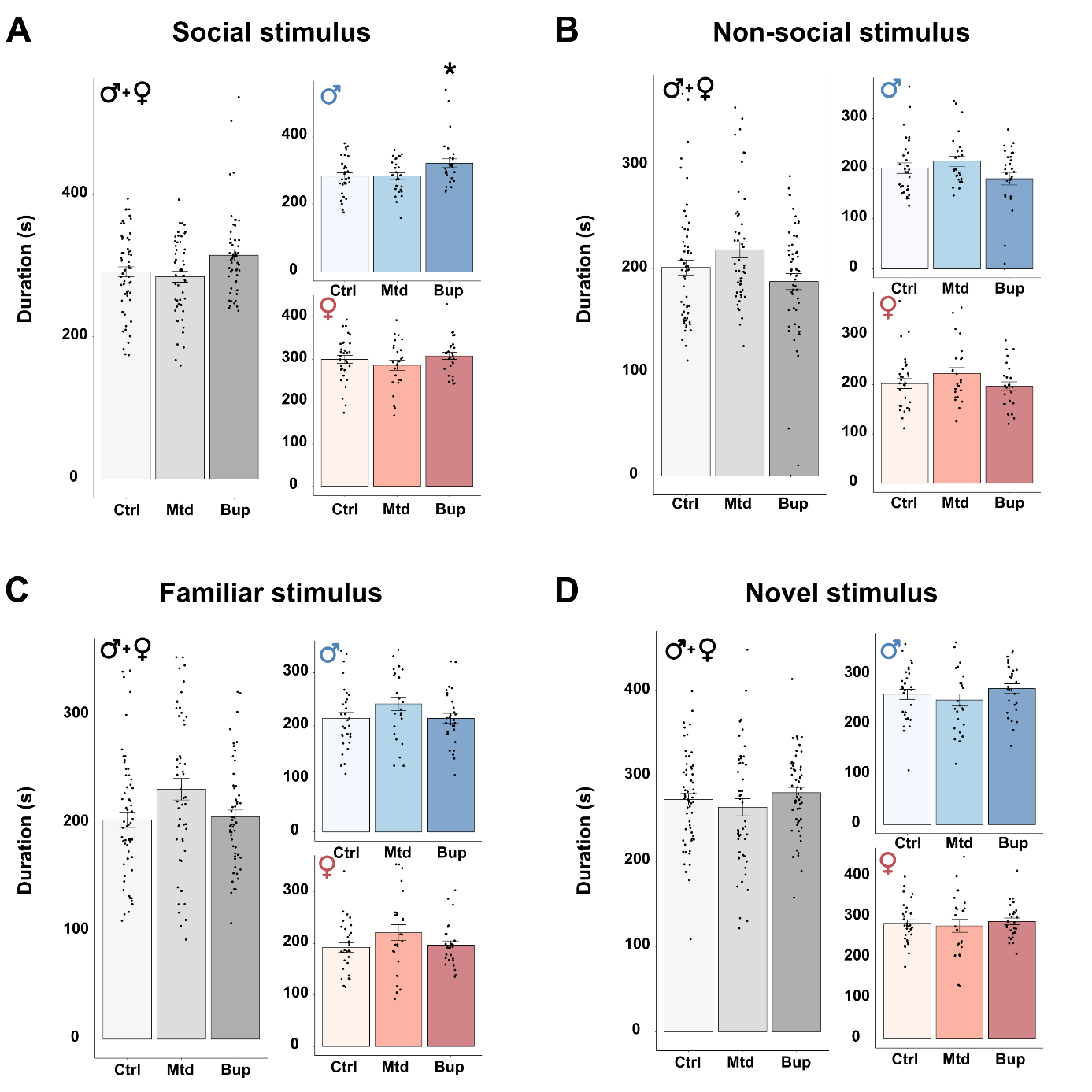 |
| --- |
| **Fig. S4** Three-chamber social interaction test in the F1 generation. Time (s) spent in the social (A) and non-social (B) chamber in the sociability phase, and time (s) spent in the chamber containing the familiar (C) and novel (D) conspecific in the social novelty phase. Values are shown as mean ± SEM, along with individual data points, n = 17-22 individuals/treatment group/sex. *p < 0.05, compared to control. Abbreviations. Bup, buprenorphine; Ctrl, control; Mtd, methadone |

| 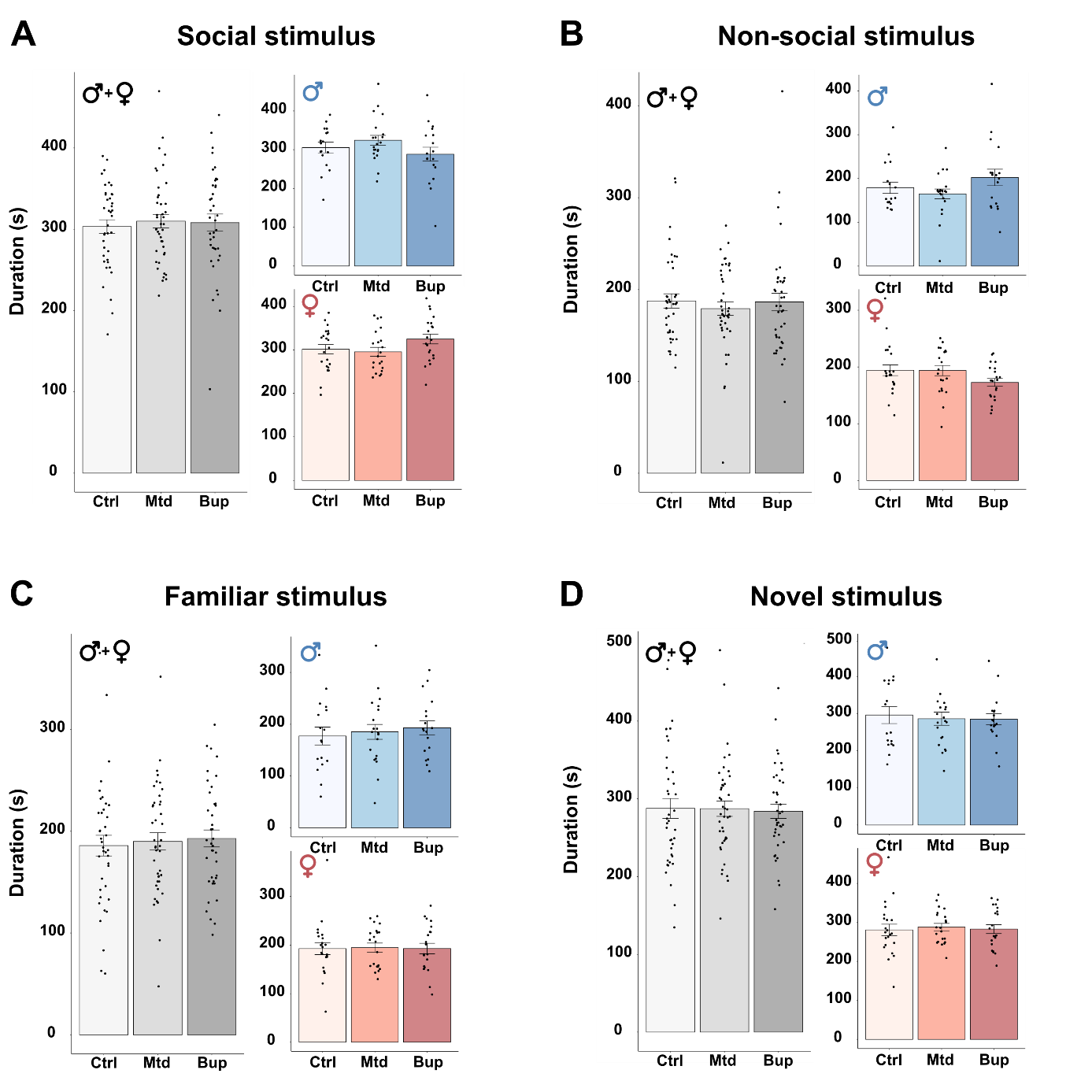 |
| --- |
| **Fig. S5** Three-chamber social interaction test in the F2 generation. Time (s) spent in the social (A) and non-social (B) chamber in the sociability phase, and time (s) spent in the chamber containing the familiar (C) and novel (D) conspecific in the social novelty phase. Values are shown as mean ± SEM, along with individual data points, n = 17 - 22 individuals/treatment group/sex. Abbreviations. Bup, buprenorphine; Ctrl, control; Mtd, methadone |

| **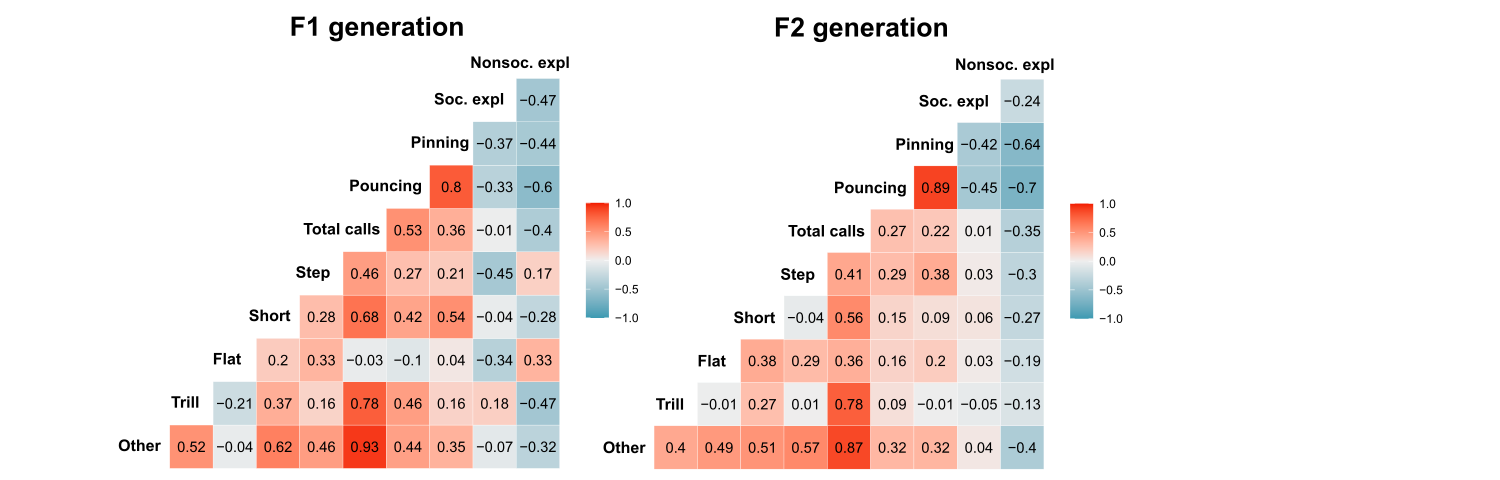 Fig. S6** Correlation between different call types and play behaviors for the F1 and F2 generations. Pearson correlation coefficients are indicated, colored from blue to red, where blue represents negative correlations, whereas red represents positive correlations |
| --- |
